# A systematic review of associations between the environment, DNA methylation, and cognition

**DOI:** 10.1093/eep/dvae027

**Published:** 2024-12-16

**Authors:** Sophie Glover, Jacob Illyuk, Claire Hill, Bernadette McGuinness, Amy Jayne McKnight, Ruth F Hunter

**Affiliations:** Institute of Clinical Science B, Royal Victoria Hospital, Centre for Public Health, Queens’ University Belfast, Grosvenor Rd, Belfast BT12 6BA, United Kingdom; Institute of Clinical Science B, Royal Victoria Hospital, Centre for Public Health, Queens’ University Belfast, Grosvenor Rd, Belfast BT12 6BA, United Kingdom; Institute of Clinical Science B, Royal Victoria Hospital, Centre for Public Health, Queens’ University Belfast, Grosvenor Rd, Belfast BT12 6BA, United Kingdom; Institute of Clinical Science B, Royal Victoria Hospital, Centre for Public Health, Queens’ University Belfast, Grosvenor Rd, Belfast BT12 6BA, United Kingdom; Institute of Clinical Science B, Royal Victoria Hospital, Centre for Public Health, Queens’ University Belfast, Grosvenor Rd, Belfast BT12 6BA, United Kingdom; Institute of Clinical Science B, Royal Victoria Hospital, Centre for Public Health, Queens’ University Belfast, Grosvenor Rd, Belfast BT12 6BA, United Kingdom

**Keywords:** systematic review, environment, pollution, metals, methylation, epigenetic, cognition, brain, neurodegenerative disease, biological pathways

## Abstract

The increasing prevalence of neurodegenerative diseases poses a significant public health challenge, prompting a growing focus on addressing modifiable risk factors of disease (e.g. physical inactivity, mental illness, and air pollution). The environment is a significant contributor of risk factors which are known to impact the brain and contribute to disease risk (e.g. air pollution, noise pollution, green and blue spaces). Epigenetics can offer insights into how various environmental exposures impact the body to contribute to cognitive outcomes. In this systematic review, we examined studies which have associated an environmental exposure to a type of epigenetic modification, DNA methylation, and a cognitive outcome. We searched four databases with keywords “environmental exposures,” “epigenetics,” and “cognition.” We yielded 6886 studies that we screened by title/abstract followed by full text. We included 14 studies which focused on four categories of environmental exposure: air pollution (*n* = 3), proximity to roads (*n* = 1), heavy metals (*n *= 6), and pesticides (*n* = 4). Overall, *n* = 10/14 studies provided evidence that DNA methylation is statistically significant in the association between the environment and a cognitive outcome. Furthermore, we identified that *n* = 5/14 studies performed a type of biological pathway analysis to determine the presence of biological pathways between their environmental exposure and cognitive outcome. Our findings underscore the need for methodological improvements and considerations in future studies, including investigation of other environmental exposures considering tissue-specificity of methylation profiles and stratifying analysis by sex, ethnicity and socioeconomic determinants of disease. This review demonstrates that further investigation is warranted, the findings of which may be of use in the development of preventative measures and risk management strategies for neurodegenerative disease.

## Introduction

### Increasing burden of neurodegenerative disease

**Figure 1.**
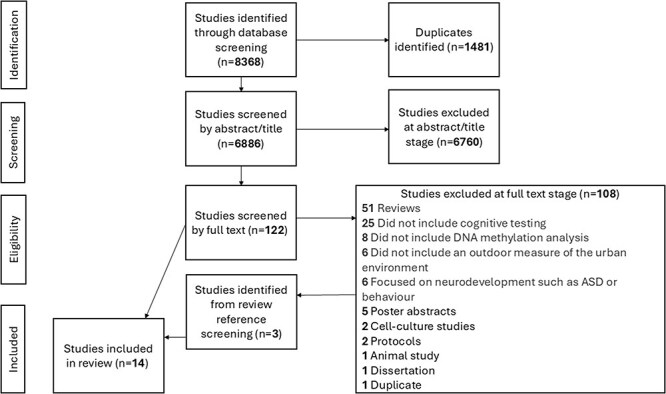
PRISMA diagram.

The burden of neurodegenerative disease, such as Alzheimer’s disease (AD), is progressively increasing, highlighted when looking at both incidence and economic figures [1]. From 1990 to 2016, there was a 117% increase in the prevalence of Alzheimer’s and other dementias globally and there was a 39% increase in deaths from all neurological disorders [2, 3]. In the 5-year period 2010–15, the global economic costs of dementia increased from US$604 billion to US$818 billion [4] with this figure projected to grow further as our population ages and disease incidence increases. Dementia is an umbrella term for neurodegenerative disorders (e.g. AD, frontotemporal dementia, lewy body dementia) in which cognition progressively declines, impacting quality of life. There are numerous known risks factors for dementia. Risk factors including age and sex [5] are nonmodifiable, while physical inactivity, mental illness (e.g. depression), and environmental exposures (e.g. air pollution) are considered modifiable [6]. Focusing research on these modifiable factors is beneficial for developing preventative strategies, enabling populations to reduce their risk of disease by altering risk factor exposure.

### Environmental risk factors

The environment (i.e. the physical and natural environment) is a significant contributor to exposing populations to multiple risk factors of neurodegenerative disease [7–9]. In the environment, individuals are chronically exposed to polluted air containing heavy metals and particulates [e.g. particulate matter (PM), lead, and cadmium], which have been associated with neurodegenerative risk [10, 11]. Other environmental characteristics such as walkability (i.e. the ability to walk to desired locations without a car [12]), cyclability (i.e. the ease with which we can cycle in a given area), accessibility, as well as the presence of green and blue space, can promote physical activity levels [13, 14]. Therefore, residents living in neighbourhoods lacking these health benefitting characteristics can experience physical inactivity, which increases neurodegenerative risk. Other environmental pollutants have been associated with increased risk of cognitive impairment (an indicator of neurodegenerative or neurological disease [15]) and neurodegenerative disease in the literature. For example, heavy metals such as cadmium and lead, present in soil and water, have been associated with cognitive impairment [16] as well as other constitutes of water pollution such as bisphenol A [17]. High levels of noise exposure are associated with progressive hearing loss, a further modifiable risk factor of dementia [6], and has implications on stress and sleep levels, suspected to be involved in biological pathways [12]. Noise pollution commonly comes from sources such as traffic and industry [18], and can potentially increase the risk of cognitive impairment and neurodegeneration. Light pollution is a further concern to populations as outdoor light at night has recently been shown to increase the risk of mild cognitive impairment [19]. Urbanization is increasing globally, with the number of people residing in urban areas expected to reach approximately 7 billion by 2050 [20]. This highlights the growing urban population, which is projected to comprise nearly 68% of the global population by 2050 [20]. Residing within urban areas where traffic-related pollution, noise pollution and light pollution for example are prevalent, can heighten residents exposure to risk factors of neurodegenerative disease, making urban environments a crucial factor to consider in disease risk.

To date, the majority of literature in the field of the environment and neurodegenerative disease has focused on investigating associations, with little research exploring the underlying mechanisms or biological pathways. It is vitally important to explore these mechanisms to understand how environmental factors impact underlying biological processes, and the mechanisms by which this could result in neurodegenerative disease. Biological pathways from the exposure to the disease state are commonly suggested to involve inflammation, a key player in neurodegenerative pathophysiology [21]. For example, air pollutant exposure such as PM2.5 [22] has been evidenced to trigger neuroinflammation and increase reactive oxygen species production, leading to cellular impairment and progressive neurodegeneration [23]. An additional mechanism, which has been explored more recently in the context of environmental exposures, is epigenetic change [12, 24–26].

The epigenome is subject to modification through environmental exposure, impacting gene expression while not changing the underlying gene sequence [27]. A commonly studied example of epigenetic modification is DNA methylation (DNAm), enzymatic additions of methyl groups to specific CpG genomic sites (regions of DNA where in a cytosine nucleotide is immediately followed by a guanine nucleotide) [28]. Exposure to environmental pollutants including air [29, 30], soil [31, 32], water [32, 33], and noise [34, 35] have been evidenced to modify the epigenome [12].

### Knowledge gaps in epigenetic literature

Epigenetic modifications relating to the environment have not been extensively investigated in the literature, particularly regarding cognitive impairment and neurodegenerative disease [12]. Several studies have found that constitutes of air pollution are associated with epigenetic markers and dementia incidence [36] or pathological marks of AD [37] for example. However, issues can arise in the current literature when there is insufficient description of the study area, particularly when researchers do not include or do not have access to longitudinal data of participant address [38, 39]. In terms of biological samples, the majority of studies have focused on analyzing methylation in blood samples despite the tissue-specific nature of epigenetic modifications [40, 41]. In a previous review, we summarized the current stance of the field addressing the environment, epigenetics, and cognition [12]. We identified that there is limited literature focusing on exploring epigenetics in the environmental–neurodegenerative relationship, meaning that we have limited biological knowledge. Most commonly, studies examining the relationship between the environment and cognition focus on air pollution, producing a lack of knowledge on other exposures such as light and noise pollution. A number of studies have explored the impact of environmental changes on epigenetics in cell culture and mouse models [42, 43]. However, there are few human studies studying this in the context of cognitive health, which also reduces our ability to explore the complexity of the impact of environmental co-exposures and limits our ability to gain insights into underlying pathological mechanisms [44, 45]. It is imperative to advance biological pathway knowledge to create a sound understanding of underlying epigenetic mechanisms and biological processes contributing to neurodegenerative pathophysiology.

It is hoped that advancing fundamental knowledge of mechanistic pathways will provide evidence to support the development of preventative strategies and policies to decrease the burden of neurodegenerative disease. Developing plans to enhance environmental health, to reduce the burden of pollutants and other environmental exposures, and implementing health-promoting environments, such as green spaces, hold potential to improve cognitive outcomes and overall health. Epigenetics may provide a link between neurodegenerative disease and the environment; it is of interest to explore modifiable and potentially reversible [46] epigenetic markers which can be readily assessed and visualized through novel analytical techniques (e.g. epigenome-wide association studies, EWAS [47]).

### Aims of the review

In summary, research regarding the biological associations between the environment and neurodegenerative disease is sparse and, to our knowledge, no other published study has systematically reviewed the literature [12]. In line with the findings of our previous review [12], there is a need to summarize current studies which have analyzed the environment, methylation, and cognitive outcomes to identify significant findings and locate knowledge gaps. Therefore, the main aims of this systematic review were to synthesize and evaluate the current findings of studies which have identified epigenetic markers (specifically DNAm), which are the consequence of environmental exposures associated with cognitive outcomes including cognitive function, cognitive impairment, and/or neurodegenerative disease. This review further aimed to conduct sub-analysis by sex, ethnicity, and deprivation, perform risk of bias assessments on all included studies and where possible identify developments in biological pathway knowledge to help us better understand disease biological processes.

## Methods

The protocol for this review was registered on PROSPERO (CRD42023424186) and produced in accordance with PRISMA guidelines [48]. There were no changes to the original protocol.

### Search strategy

Four databases (PubMed, EMBASE, PsycINFO, and Medline) were searched from the database start date to 25 September 2023 for human studies written in the English language. Additional searches were performed in bioRxiv and medRxiv in September 2022–September 2023 for preprint articles. Searches were performed linking the three main concepts “epigenetic,” “environmental exposures,” and “cognition” to identify studies that have investigated the association between an environmental exposure (air, soil, water, light, or noise pollution, green or blue space, walkability, cyclability, temperature, or more novel exposures including microplastics and wildfires) with DNAm and cognitive function, cognitive impairment, or neurodegenerative disease. We additionally search the term “urban environment” considering the significant role of urban environments in contributing to exposure levels or likelihood. Search terms were generated based on variables included within a Causal Loop Diagram (CLD) exploring the inter-relationships between urban environment factors and neurodegenerative disease [49] and discussed within Glover *et al*.’s, [12, 50] reviews, with refinement by co-authors of this review. The search strategy and PICO criteria for this review can be found in Supplementary Files Section 1 and the definitions of the main concepts in this review can be found in Supplementary Files Section 2.

### Eligibility criteria

The eligibility criteria are outlined in Table 1. Importantly, for a study to be included in this review, an environmental exposure of interest had to be associated with an epigenetic marker (DNAm) of cognitive function, impairment, or neurodegenerative disease through formal association analysis. Studies which used cognitive function as their cognitive domain as opposed to cognitive impairment or neurodegenerative disease were included in this review for several reasons. It is becoming increasingly apparent that risk factors and exposures from younger life are impacting our health and disease outcomes in later life [51, 52]. Environmental exposures associated with differential methylation profiles in early life and lower cognitive function are of interest to the field. Further analysis of these markers and their role in biological pathways that may contribute to declines in cognitive function may elucidate future risk of cognitive impairment and associated biological pathways. Studies which specifically used testing of neurodevelopmental outcomes as opposed to cognitive outcomes or used mental health outcomes were not included in this review. Studies which used a diagnosis of a neurodegenerative disease according to clinical criteria were included in this review, the diagnosed neurodegenerative disease being classified as their cognitive outcome.

**Table 1.** Eligibility criteria

Inclusion	Exclusion
Human population-based quantitative study	Cell culture/*in vitro* study
Studies which included participants of any age, sex, or from any country	Animal studies
Studies investigating one of the following environmental exposures/characteristics: 1. Outdoor air pollution including particulate matter, ozone, sulfur oxides, nitrogen oxides, carbon oxides, heavy metals 2. Outdoor soil pollution including heavy metals, chemicals, or pesticides 3. Outdoor water pollution including heavy metals, phenols, or other chemicals 4. Outdoor noise pollution from sources including transport, industry, construction 5. Outdoor light pollution focused on outdoor light at night and streetlight density 6. Green or blue space access and quality including parks, greenways, public gardens, public open space, park rivers or lakes, trees, and low-lying vegetation 7. Neighbourhood walkability measured by land use mix, green space presence, pedestrianisation, traffic density, sidewalk density 8. Neighbourhood cyclability (bikeability) measured by cycle lane availability 9. Heat/temperature measures by heatwave statistics or longitudinal temperature measurement 10. Novel exposures/pollutants including microplastics, wildfires, and forest fires [12, 49, 50]	Studies which associated/investigated only two out of three of the main concepts of this review (i.e environment and DNAm OR DNAm and cognitive impairment)
Studies which associated an environmental exposure of interest with cognitive function, cognitive impairment or neurodegenerative disease through DNAm analysis	Studies which investigated histone modification or non-coding RNA as opposed to DNAm
Studies which assessed global DNAm	Studies investigating genetic markers as opposed to epigenetic (DNAm) markers
Preprint articles	Theses, conference abstracts, protocols, or dissertations
	Review articles

### Screening

The search strategy yielded 6886 studies to be screened once duplicates were removed (Fig. 1). Identified studies were exported to the Covidence software for screening. Two independent reviewers (S.G. and C.H./J.I.) screened studies by title/abstract followed by full text. Any disagreements were resolved with the aid of a third independent reviewer (A.J.M. or R.H.). Additionally, relevant review articles published from 2021 to 2023 were subjected to reference list screening, along with any review article which was identified as particularly relevant. Overall, 14 studies met the predetermined eligibility criteria. Eight studies investigated adult (aged >18 years) cognitive impairment or neurodegenerative disease while six studies focused on children’s (aged <18 years) cognitive function. Children focused on studies which discussed neurodevelopment while using tests of child cognitive function were included in this study, for example Lee *et al*.’s [53] and Huen *et al*.’s, [54] studies, which used the Wechsler Intelligence Scale for Children (WISC).

### Extraction

Data were extracted by two independent reviewers (S.G. and C.H./J.I.). The extracted data included: study authors, year, location, study design, population/cohort description (sample size, age, sex, indicator of deprivation, ethnicity, and time of data collection including follow-up period), phenotype description (testing of cognitive function, diagnosis of cognitive impairment or neurodegenerative disease, and stage of disease), epigenetic marker specifications (DNA methylation quantification/identification assay), type of statistical analysis, relevant statistical outcomes (M values, B values, hazard/odd ratios, relative risk, *P*-values, and effect sizes), outcomes (main and stratification by sex, deprivation, and ethnicity), biological pathway information (evidence from within study pathway-based analysis). Extracted data from included studies can be found in Tables 2**–**6 .

**Table 2.** Data extraction—air pollution

Author/year	Country/income status	Study design	Study characteristics	Time of data collection (incl follow up)	Cognitive descriptions	Generation of DNA methylation	Environmental exposure	Type of association analysis	Covariates	Outcome: main analysis	Outcome: sex
Zhenjiang Li/2023 [55]	USA (high)	Cross-sectional	Sample size: 159 * Mean age: 76.6. years Deprivation measure: Deprivation Index mean 36.3 78.7% college education or more and living in less deprived neighbourhoods Ethnicity: 89.3% White 10.7% Black Sex: 56%M, 44%F 95.6% diagnosed AD or another form of dementia, 56% at least one *APOE4* allele	2005-2020 ADRC cohort recruitment 2002–19 Air Pollution (PM2.5) No follow up	AD-related neuropathological changes were evaluated using Braak stage, CERAD score, and a combination of Amyloid, Braak stage, and CERAD (ABC) score which were developed based on the Aβ plaques and NFTs	Sample: prefrontal cortex. DNA isolation: QIAGEN GenePure kit. DNAm: Illumina Infinium MethylationEPIC. BeadChips in batches of 167 samples with 6 replicates. 159 samples passed QC, 789 286 CpG sites remained after exclusion of low-quality probes for example	Specific exposure: Traffic-related PM2.5 Measurement: Statistical models were separately trained to estimate exposures for 2002–11 and 2012–19, respectively. For example, the 2012–19 exposure model was trained in an R package using accumulated data from Atlanta Regional Commission, Georgia Department of Transportation, National Land Cover Database and the Atmospheric Composition Analysis. This model predicted traffic-related PM2.5 within a 200 metre spatial resolution Calculated for 1 year, 3 years and 5 years pre death by matching residential address to closest calculated PM2.5 grid Environmental context: Participants included from any residential address in Atlanta, Georgia Exposure time/length: NR	EWAS for long-term PM2.5 exposure 1, 3, and 5 years prior to death with multiple linear regression (Bonferroni significance = 6.33 × 10^−8^). Significant CpGs Ffrom EWAS then associated with neuropathic CpGs (from EWAS of each pathological mark: CERAD, Braak stage, ABC score) by multiple linear regression. Combined MITM with HDMA to identify CpGs which did not reach genome-wide significance.	Sex, race, educational attainment, age of death, *APOE4* genotype, Area Deprivation Index estimate, post-mortem hours of sample collection estimate Minimum adjustment for the association between PM2.5 exposure and DNA methylation = sex, race, age at death, educational attainment, and ADI, PMI, and cell-type proportions. The minimum adjustment for the association between DNA methylation and neuropathology markers = sex, race, age at death, educational attainment, *APOE E4* genotype, PMI, and cell-type proportions.	Outcome of Interest: Four overlapping CpGs identified in the MITM: cg01835635 (*APOA4*) associated with CERAD and PM2.5 (1- and 3-year exposure). cg09830308 (*MLKL*) associated with Braak stage and PM2.5 (1-, 3-, and 5-year exposure). cg16342341 (*SORBS2*) associated with CERAD and 1 year PM2.5 exposure. cg27459981 (*MLKL*) associated with Braak, ABC, and PM2.5 (3- and 5 year exposure) Other Outcomes: cg25433380 (c9) and cg10495669 (c20, *RBCK1*) associated with PM2.5 after correction (e.g. *P* = 1.58 × 10 ^− 8^ for 1 year pre-death PM2.5 exposure for cg25433380). HDMA with DACT and causal mediation analysis identified 22 CpGs mediating the association between PM2.5 and ABC score. *SORBS2* was associated with all three exposure windows.	NR
Joan Lee/2017 [53]	China (upper–middle)	Cohort	Sample size: 305 mother/infant pairs *N* = 148pairs 2002 *N* = 157 pairs 2025 Mother’s living within 2.5 km of Tongliang power plant, nonsmoking, mothers >20 years of age. Mean age: mother’s age: 2002 cohort—mean 25.27 years 2005 cohort—mean 27.8 years Children 5 years at cognitive testing Deprivation measure: mother’s education: 2002 cohort—51.68% high school or above 2005 cohort—42.68% high school or above Ethnicity NR Sex: Children 2002 cohort 74%F Children 2005 cohort 45.22%F	Cohort enrolment 2002 or 2005	WISC at age 5 years	Sample: umbilical cord blood collected at delivery (buffy coat then separated). Bisulfite treatment: EZ-96 DNA methylation-lightning kit (ZYMO Research), LINE1 methylation at three CpGs in the promotor region by PCR of bisulfite DNA and pyrosequencing	Specific exposure: PAH exposure from coal power plant—closed in 2004 so samples collected 2002 vs 2005 to identify differences Measurement: PAH-DNA adducts measures from maternal (1-day postpartum) and umbilical cord white blood cell DNA by a high-performance liquid chromatography /fluorescence method Environmental context: all mothers were living within 2.5 km of the Tongliang coal-fired power plant located in Tongliang city. The power plant closed in 2004 (therefore cohort recruitment before and after to identify biological impacts) and was stated to be the principal source of air pollution in the city. Exposure length/time: NR	*T*-test for cohort comparative analysis Multivariate linear regression to look at the association between PAH-DNA adducts and LINE1 methylation and between LINE1 methylation and IQ score	Association between PAH-DNA adduct and LINE1 methylation levels—Mother’s age, mother’s education, household income, child gender. Regression analysis for LINE1 methylation and IQ scores—cord lead levels (Ln), gender, gestational age, and mother’s education	Outcome of interest: LINE1 methylation did not mediate the relationship between PAH-DNA adducts and IQ scores. Other outcomes: Significant differences in LINE1 methylation between cohorts: hypermethylation in 2005 (a = 0.05 in t test). PAH-DNA cord adduct level inversely associated with LINE1 methylation (β = − 0.010, *P* = .033, *N* = 217). LINE1 methylation positively associated with child IQ in WISC Full Scale (β = 85.304, *P* = .005) and verbal testing (β = 94.362, *P* = .003) (2002 cohort, children 5 years of age)	NR
Jia Guo/2022 [56]	USA (high)	Cohort	Sample size: 341 mother/infant pairs, for which cord sample DNAm data was generated for (Columbia Centre for Children’s Environmental Health) (*N* = 240 random discovery set, *N* = 101 remaining for validation set). Mean age: Mother’s age mean 24.5 years Children aged 5 and 7 years during cognitive testing Deprivation measure: 45% had annual household income <$10 000 Ethnicity: Mother’s 43% African American, 57% Dominican Sex NR	1998-2006 cohort enrolment	FSIQ of the (WISC-IV) at age 5 and 7	Sample: umbilical cord blood DNAm: 450 K array (485 577 CpG sites) and the EPIC array (866 895 CpG sites)	Specific exposure: BPA, CPF, the sum of phthalate DEHP metabolites, PBDEs, PAH, PAH-DNA adducts in maternal and cord blood, PM2.5, and NO_2_ Measurement: PAH and PAH adducts—raw measurements from maternal urine PM2.5 and NO_2_—average daily measurements throughout gestational period Environmental context: All recruited mothers lived in Northern Manhattan or South Bronx for at least a year before pregnancy Exposure time/length: NR	Random discovery set (70% of 341 samples) and validation set (remaining 30%). For each neurodevelopmental outcome, keywords were defined and related biological pathways identified via the KEGG database. CpG sites within these genes identified. For each outcome, associations tested with each preselected CpG. For each outcome, associations between each of the environmental exposures and each of the significant CpGs were tested. For each outcome, they tested associations with environmental exposures. Linear regression models with adjustment to associate CpGs with cognitive test scores. Significant CpGs from this were then associated with environmental exposures. FDR with adjusted *P* < .05 as the threshold to correct for multiple comparisons. Mediation analysis with R MMA package.	For each outcome, associations tested with each preselected CpG- Infant sex, ethnicity, infant age of testing when outcomes measured For each outcome, associations between each of the environmental exposures and each of the significant CpGs tested—children’s sex and ethnicity. For each outcome, they tested associations with environmental exposures—children’s sex, ethnicity, and children’s age of testing.	Outcome of interest: No significant CpGs for FSIQ age 5 or 7.	NR

AB = amyloid beta; ABC = Amyloid, Braak stage, and CERAD; AD = Alzheimer’s disease; ADRC = Alzheimer’s Disease Research Centers; ANOVA = analysis of variance; APOE4 = apolipoprotein E4; BMI = body mass index; CAS = Cognitive Abilities Screening; CASI = Cognitive Abilities Screening Instrument; CERAD = Consortium to Establish a Registry for Alzheimer’s Disease; CHAMACOS = Center for the Health Assessment of Mothers and Children of Salinas; CPF = chlorpyrifos; CRTC = Combined Raven’s test for China; CRT-C2 = Chinese Revised Token Test, revised in 1997 for the second time; DACT = Divide-Aggregate Composite-null Test; DAP = diammonium phosphate; DE = diethyl; DEPH = Di(2-ethylhexyl)phthalate; DIG-PD = drug interaction with genes in Parkinson’s disease; DM = dimethyl; DMR = differentially methylated region; DNAm = DNA methylation; DNAmAge = DNA methylation age; DML = differentially methylated loci; EWAS = epigenome-wide association study; F = female; FDR = false discovery rate; FSIQ = Full-Scale intelligence Quotient; GIS = geographic information systems; GRS = genetic risk score; HDMA = high-dimensional mediation analysis; HSD = honest significant difference; IQ = intelligence quotient; M = male; MCI = mild cognitive impairment; MMSE = mini mental state examination; mQTL = DNAm quantitative trait loci; MR = Mendelian randomization; NA = not applicable; NFT = neurofibrillary tangle; NO = nitric oxide; NR = not reported; OGC = organochlorine; PAH = polycyclic aromatic hydrocarbons; PBDE = polybrominated diphenyl ethers; PCA = principle component analysis; PD = Parkinson’s disease; PEG = Parkinson’s environment and genes; PM = particulate matter; PPVT = Peabody Picture Vocabulary Test; QC = quality control; SGPD = System genomics of Parkinson’s disease; UPDRS = Unified Parkinson’s Disease Rating Scale; WISC = Wechsler Intelligence Scale for Children; WRAML = Wide Range Assessment of Memory and Learning; WRAVMA = wide range assessment of visual and motor abilities.

**Table 3.** Data extraction—proximity to roads

Author/year	Country/income status	Study design	Study characteristics	Time of data collection (incl follow up)	Cognitive descriptions	Generation of DNA methylation	Environmental exposure	Type of association analysis	Covariates	Outcome: main analysis	Outcome: sex
Cheng Peng/2018 [57]	USA (high)	Cohort	Sample size: 482 mother/infant pairs (Project VIVA) Mean age: Mother’s age at enrolment mean 32.1 years Children age at cognitive testing NR Deprivation measure: Median household income ($1000) mean 57 Ethnicity: Mother’s 71% White, 12% Black, 17% Other Sex: Children 52% M 48% F	1999–2002 cohort enrolment	KBIT, second edition (KBIT-2), visual memory index of the WRAML, second edition (WRAML2), visual-motor subtest of WRAVMA during mid-childhood visit	Sample: umbilical cord blood DNA extraction: Qiagen Puregene® Kit (Qiagen, N.V.) Bisulfite conversion: EZ DNA Methylation-Gold™ Kit (Zymo Research) DNAm: Infinium® HumanMethylation450 BeadChip (Illumina, Inc.)	Specific exposure: Traffic-related exposures from proximity to road Measurement: Residential address at birth based on maternal-self reported questionnaire. Residential proximity to major roads (A1—primary highway, limited access and A2—primary road without limited access) using geocoded address and ArcGIS 10.1 Street Map™ North America (ESRI) Mean distance to major A1/A2 was 1881.2 m Environmental context: All mothers lived within proximity to visit the Atrius Harvard Vanguard Medical Associates in eastern Massachusetts Exposure time/length: NR	Linear regression for associations between log-transformed residential proximity to roadways and DNAm. FDR-corrected *P*-value of *P* < .05 based on the method of Benjamini and Hochberg. As a secondary analysis, they used a regional-based approach to identify differentially methylated regions associated with prenatal residential proximity to major roadways. *P*-value cutoff of 10^−08^, with Benjamini–Hochberg (BH) adjustment. Multivariate linear regression model to look at *LAMB2*-related CpGs in cord blood with cognitive tests (FDR < 0.05).	Methylation associations—maternal characteristics [age at enrolment, pre-pregnancy body mass index, race/ethnicity, smoking status, education level, neighborhood income, child characteristics [gestational age at birth, sex, season of birth], and estimated cord blood cell proportions [percentages of monocytes, CD8T cells, CD4T cells, NK cells, B cells, and nucleated red blood cells] *LAMB2* association—Maternal age at enrolment, maternal ethnicity, maternal smoking status, maternal education level, child age at cognitive test and child sex.	Outcome of interest: Overlapping significant CpGs inversely associated with methylation in KBIT-2 nonverbal test: cg05654765 (−2.74 points, 95% CI (−5.29, −0.20), *P* = .03), cg14099457 (−2.28 points, 95% CI (−4.57, 0.00), *P* = .05), cg03732535 (−10.89 points, 95% CI (−17.85; −3.94), *P* = .002), and cg02954987 (−1.45 points, 95% CI (−3.00, 0.11), *P* = .07). Other Outcomes: Four CpGs within *LAMB2* associated with living closer to major roadways at birth (PBH < 0.05). Increased DNA methylation with halving residential proximity to major roadways: cg05654765 (0.82%, 95% CI (0.54%, 1.10%), *P* = 2.4^e–10^), cg14099457 (0.88%, 95% CI (0.56%, 1.19%), *P* > .05), cg03732535 (0.19%, 95% CI (0.11%, 0.28%), *P* = 4.8^e–07^), and cg02954987 (1.08%, 95% CI (0.65%, 1.51%), *P* = 6.0^e–07^).	NR

**Table 4.** Data extraction—heavy metals

Author/year	Country/income status	Study design	Study characteristics	Time of data collection (incl follow up)	Cognitive descriptions	Generation of DNA methylation	Environmental exposure	Type of association analysis	Covariates	Outcome: main analysis	Outcome: sex
Susan Searles Nielsen/2015 [58]	USA (high)	Nested case–control	Sample size: 201 Age range: 25–65 years Deprivation measure: >12 years education: 24% of UPDRS3 ≥ 15, 23% of UPDRS3 > 8 to ≤ 12, 20% of UPDRS3 < 6 (control) Ethnicity: 100% Non-Hispanic Caucasian Sex: 100% M	2006–13 cohort collection	A neurologist specialising in movement disorders examined participants using a standardized exam including the UPDRS subsection 3 (UPDRS3). UPDRS3 < 6 (control, *n* = 103) UPDRS3 >8 to ≤ 12 (intermediate, *n* = 49) UPDRS3 ≥ 15 (Parkinsonism, *n* = 49)	Sample: whole-blood DNA isolation: QIAamp DNA blood kit Bisulfite treatment: Qiagen’s EpiTect Fast DNA Bisulfite Kit, DNAm: status determined by Pyromark Q24 NOS2 methylation at three CpGs was assessed (in line with a previous study on welders)—sites 8309, 8314, 8329	Specific exposure: Welding fumes hypothesized to contain high heavy metal concentration such as Manganese Measurement: Self-reported exposure to welding fumes Participants were workers or retirees of one of three welding sites—two shipyards and one heavy equipment fabrication shop Environmental context: All participants were a part of an ongoing study in Midwest USA Exposure time/length: Cumulative duration of occupational exposure with self-reported work histories (mean 19.8 years)	Logistic regression models to compare parkinsonism and intermediate UPDRS3 to controls. %*NOS2* methylation in the primary analysis equated to mean percent methylation across the three CpGs of interest (In secondary analysis, %methylation was at individual sites) Linear regression to look at the association between CpG 8329 and welding exposure	All models adjusted for examiner, age, and experimental plate. Other considered confounders didn’t alter results so were not included in models. Sensitivity analyses addressed co-morbidities (stroke was in the exclusion criteria for the original cohort)	Outcome of interest: All controls had >90% methylation of each CpG. Inverse association between *NOS2* methylation and Parkinsonism after adjusting for age, examiner, and experimental plate (*P* = .04). Hypomethylation associated withParkinsonism. CpG 8329 showed significant associations when excluding retirees (*P* = .01). Each 1% increase in CpG site 8329 methylation in non-retired workers associated with a 33% lower prevalence of Parkinsonism (95% CI: 8%, 51%). Other outcomes: No significant associations were found in linear regression models for welding exposure and CpGs after adjusting for age and experimental plate. Workers with recent exposure to welding fumes had 0.72% lower absolute methylation of CpG 8329 compared to retirees (CI −1.66, 0.23). Stratification by exposure duration: <10 years of exposure: 0.16% lower methylation of CpG 8329 per year (95% CI: 0.02% to 0.29%, *P*-value for trend = .03). No significant association in workers with ≤10 years of exposure (0.004%, 95% CI: −0.03% to 0.04%, *P*-value for trend =.81).	NA
Xiojuan Yang/2015 [59]	China (upper-middle)	Cross-sectional	Sample size: 366 Age range: 40–60 years Mean age: 44.9 years Deprivation measure: Education (years) No significant difference between groups based on different Aluminum serum levels, average years 10.2–10.4 Ethnicity NR Sex: 100% M	NR	MMSE (cut-offs adjusted for age and education) MCI diagnosis made by professional clinicians. Diagnostic criteria for cases outlined in study.	Sample: whole blood DNA isolation: Flexigene Kit Global DNAm: Imprint Methylated DNA Quantification Kit (Epigentek Ground Inc, NY)	Specific exposure: Aluminum from occupational exposure Measurement: Calculated cumulative duration of occupational exposure with self-reported work histories. All participants had regular aluminum exposure. Measured serum aluminum in blood samples Serum aluminum median concentration 48.99 μg/l Environmental context: : All study participants were from the same region in Zunyi, China Exposure time/length: Duration of exposure mean 21.2 years	Shapiro–Wilk normality test for distributions One-way ANOVA to compare MMSE among groups of different cognitive domains (i.e. 0–34.02 MMSE score, 34.03-61.42 MMSE score) Chi-squared to compare self-reported smoking, drinking, MCI rate and DNAm among cognitive groups T-test to look at DNAm differences between MCI and non-MCI groups Multivariate logistic regression to look at the association between MCI and global DNAm	Multivariate adjusted for cumulative time of exposure, serum Aluminum concentration, age, education, smoking and drinking	Outcome of Interest: Increased aluminum concentration correlated with decreased global DNAm. Higher serum aluminum group (>61.43) had significantly decreased global DNAm compared to lower group (0–34.02) (*P* < .05). MCI associated with: Increased risk of low global DNAm (OR 3.66, 95% CI 1.8-7.42, *P* < .001). High serum aluminum levels (OR 1.76, 95% CI 1.19–2.62, *P* = .005).	NA
Sebastian Castillo/2017 [60]	Chile (high)	Case–control	Sample size: High mining activity city: case = 45 control = 52. Low mining activity city: case = 52 control = 54. Mean age: High mining activity city: case mean 72 years, control mean 63 years. Low mining activity city: case mean 65 years, control mean 60 years. Deprivation measure NR. Ethnicity NR. Sex: High mining activity city: case 51% M, control 30.1% M. Low mining activity city: case 63.5% M, control 46.3% M.	NR	PD diagnosis by one of two neurologists based on UK Parkinson’s Disease Society Brain Bank Clinical Diagnostic Criteria	Sample: blood, DNA isolation: Tissue DNA Kit (D3396-02, EZNA), DNA quantification: El NanoDrop® ND-1000 spectrophotometer, DNAm: Methylamp Global DNA Methylation Quantification Ultra Kit (Epigenetik, catalog number P-1014B, USA), Bisulfite treatment: EZ DNA Methylation-Lightning™ Kit (Zymo Research)	Specific exposure: Heavy metals from exposure to high vs low levels of mining activity Measurement: Living in a city with high vs low mining activity Participants commented on if they were directly or not directly exposed to metals Environmental context: Antofagasta is a city with high mining activity in the north of Chile accounting for 51% of the country’s mineral production (copper, lithium, natural nitrates, iodine, molybdenum, gold, silver, and borates) while Santiago is a city with low mining activity, accounting for 0.7% of mineral production Exposure time/length: NR	ANOVA for assessing differences between mean % methylation Tukey HSD to compare mean methylation of each group in pairs to show differences between groups	NR	Outcome of interest: Significant decrease in global DNAm in cases exposed vs. not directly exposed to mining activity compared to controls in the high mining exposure city (*P* < .001). Other outcomes: Significant decrease in DNAm in cases from both cities compared to controls (*P* < .001). DNAm in controls from the mining city was decreased compared to controls from the city with less mining activity (70%, *P* < .001). No significant difference in global DNAm in cases from cities, regardless of direct exposure to metals in the high mining activity city.	NR
Kimberly Paul /2021 [61]	Australia, New Zealand, USA (high)	Case–control	Sample size: SGPD—959 case, 930 control. PEG—569 case, 238 control. Mean age: SGPD—biological age estimated by Horvath clock—case mean 66.5years, control mean 64.9 years. PEG—case mean 70.5 years, control mean 67.5 years. Deprivation NR. Ethnicity: SGPD all European ancestry. PEG European fractional ancestry case 0.87 control 0.92. Sex: SGPD—case 63.7% M, control 46% M. PEG—case 62.6% M, 53.4% M.	NR	SGPD—PD diagnosis through a range of clinical and cognitive tests PEG—Idiopathic PD diagnosis within 3 years of recruitment, assessed against a range of clinical criteria	Sample: whole blood DNAm: Human Methylation 450 k BeadChip for genome-wide DNAm	Specific exposure: Generated two epigenetic biomarkers for cumulative lead exposure (tibial (138 CpG) and patella (59 CpGs). This was developed in the Normative Aging Study Measurement: Long-term exposure to lead—study states common occupational lead exposure and chronic lead exposure from drinking water. Environmental context: SGPD is based on participants from across Australian and New Zealand PEG is based on participants from three agricultural counties in Central California Exposure time/length: NR	Logistic regression to explore relationship between DNAm lead and PD Cochran’s Q for between-study heterogeneity with a fixed effects model	Age (Horvath DNAmAge in SGPD), sex, ancestry (PEG only), blood cell composition, smoking history (PEG only), and mean methylation by sample to account for global methylation	Outcome of interest: PD status strongly associated with DNAm biomarker for tibia-lead levels (OR 1.52 [1.25, 1.86], *P* < .0001; no significant heterogeneity). PD status inversely associated with DNAm patella-lead levels in SGPD (OR 0.7 [0.53, 0.93], *P* = .015) but not in PEG.	DNAm tibia lead significant in both women and men in both cohorts DNAm patella lead significant when stratified by sex in SGPD, i.e. same results
Andres Cardenas/2017 [62]	USA (high)	Cohort	Sample size: 321 mother/infant pairs Longitudinal analysis in 70 children with blood samples in early childhood and 160 children with blood samples mid-childhood (43 had both childhood blood samples) From Project VIVA Mean age: Mother’s age at enrolment mean 31.9 years Early childhood mean 3.4 years Mid-childhood mean 7.9 years Deprivation measure: Mothers—67% college graduates Ethnicity: 77.3% mothers White, 8.7% Black, 6.5% Hispanic, 7.5% Other. 72.9% children White, 9.75 Black, 4.4% Hispanic, 13.1% Other Sex: Children 49.8% M 50.1% F	1999–2000 cohort enrolment. Follow up when children 2.9–4.9 years (early childhood) and 6.7–10.5 years (mid-childhood)	PPVT and WRAVMA cognitive tests at early-childhood visit	Sample: umbilical cord blood (collected at delivery), whole blood from early and mid-childhood. Buffy coat used for DNAm assessment. Bisulfite conversion: EZ DNA Methylation-Gold Kit (Zymo Research, Irvine, CA) DNAm: Infinium Human Methylation450 BeadChip (Illumina, San Diego, CA) Cell type composition was estimated in cord blood using a reference panel of nucleated cells isolated from cord blood (leukocytes and nucleated red blood cells) and an adult leukocyte reference panel for blood samples collected in early or mid-childhood.	Specific exposure: Mercury Measurement: Red blood cell Mercury from maternal blood samples (2nd trimester) from fish intake and industrial/urban exposure Samples measured for total mercury using Direct Mercury Analyzer 80 (Milestone Inc., Monroe, Connecticut) Food survey on fish intake Mean maternal RBC Mg 3.8ng/g Environmental context: All mothers lived within proximity to visit the Atrius Harvard Vanguard Medical Associates in eastern Massachusetts Exposure time/length: NR	Regional analyses—Association of prenatal mercury exposure with DMRs in cord blood (DMRcate), adjusted for multiple comparisons using an FDR < 0.05. Linear regression models to estimate associations between DMR or CpGs in cord blood and cognitive test scores	Regional analyses—adjusting for child gestational age at delivery, sex, and estimated nucleated cell types in cord blood, maternal age, race/ethnicity, mean weekly fish intake during pregnancy, pre-pregnancy BMI, smoking during pregnancy, parity and college education. Maternal education at study enrolment, maternal PPVT, self-reported alcohol use during pregnancy, fetal growth, mean weekly fish intake during pregnancy, child’s age in days during testing, sex, child race, parity and maternal smoking during pregnancy	Outcome of interest: 4 CpGs in the *PON1* DMR (associated with prenatal mercury exposure) linked to cognitive scores in early childhood for males. Inverse multivariate adjusted association between DNAm and PPVT scores for: cg05342682 (β = −3.2, 95% CI: −6.2, −0.2) cg21856205 (β = −4.5, 95% CI: −8.7, −0.1) cg17330251 (β = −1.9, 95% CI: −3.6, −0.1) cg01874867 (β = −2.0, 95% CI: −3.7, −0.2) No significant associations for other cognitive tests in males or for females. Other Outcomes: Prenatal mercury exposure associated with hypomethylation of 9 CpGs in the *PON1* gene. Overall mean cord methylation of the 9 CpGs inversely associated with maternal RBC Mercury (β = −2.4%, 95% CI: −3.8, −1.0; *P* = 7.5 × 10^−4^). Other CpGs also associated with prenatal mercury exposure in males.	Main analysis sex-stratified
Cong Wan/2021 [63]	China (upper-middle)	Cross-sectional	Sample size: 333 (210 exposure group vs. 123 control group) Mean age: exposure group: mean 9.93 years Control group: mean 9.62 years Deprivation measure: Family annual income: Exposure group—45.4% <30k Renminibi Control group—68.5% <30k Renminibi Ethnicity NR Sex: exposure group: 46.7% M 53.3% F Control group: 48% M 52% F	NR	CRTC, revised in 1997 for the second time (CRT-C2)	Sample: blood DNA isolation: QIAamp DNA Blood Mini Kit (Qiagen, Germany) Bisulfite and DNAm: Liquid Hybridization Capture-Based Bisulphite Sequencing, differentially methylated regions confirmed with MethylTarget	Specific exposure: Heavy metal exposure from living in proximity to a steel mill vs not (namely arsenic, cadmium, mercury and lead) Measurement: Exposure group—children living in a town containing a steel mill vs Control group—children living in a neighbouring town without a steel mill. Blood samples were analyzed in all participants for arsenic, cadmium, mercury and lead with NanoDrop 2000 spectrophotometer (NanoDrop Technologies, Wilmington, DE) and 1% agarose gel electrophoresis Environmental context: Participants were recruited from two neighbouring towns within the same city in China, one with a steel mill (industrialized area) and one without Exposure time/length: NR	Differential methylation of cytosines (DMCs) was calculated by dividing the number of methylated reads covering a cytosine by all reads covering it. The DMR was calculated by averaging the differential methylation of all loci within a region. DMCs between the exposure and control groups—Mann−Whiney U (MW-U) test using p < 0.05 and average methylation difference >0.2. DMRs between exposure and outcome—MW-U test using CpG numbers in a DMR ≥ 5; distance between two adjacent loci ≤300 nt; and methylation difference >0.1 with false discovery rate (FDR)-adjusted *P*-value <.05). The differential methylation of a locus was calculated by dividing the number of methylated reads covering a locus by all reads covering it. MW-U test used with *P* = .05 as the threshold. Adjusted multivariate linear regression to identify associations between blood lead levels, intelligence and DNAm. Mediation analysis—four-way decomposition mediation analysis (decomposing to effect on exposure four ways)	Children’s age, gender, passive smoking of the children (yes/no), family annual incomes, father’s and mother’s age, occupations and education levels, premature delivery (yes/no), and delivery method (eutocia/caesarean)	Outcome of interest: Mediation analysis identified DNAm role in lead-IQ relationship *FAM50B1* and *PTCHD3* significantly mediated lead-IQ relationship (contribution rates: 30.36% (β = −0.03, *P* = .010) and 60.36% (β = −0.04, *P* < .001), respectively) Decreased lead exposure associated with hypomethylation of gene regions and lower IQ. Other Outcomes: Blood lead median: Exposure group 71.63 μg/l vs. control 37.03 μg/l (*P* < .001). Intelligence scores significantly lower in exposure group compared to controls (*P* = .032). Blood lead levels negatively correlated with intelligence (β = −0.12, 95% CI: −0.17, −0.06, *P* < .001). Significant reductions in IQ scores in higher blood lead quartiles compared to lowest quartile. DNAm of *FAM50B1* decreased significantly in higher lead quartiles compared to lowest quartile (*P* = .001, 0.001, and <.001). DNAm of *PARD6G2*, *PTCHD3*, and *WDR27* decreased significantly in highest lead quartile compared to lowest quartile (*P* = .003, .002, and .001).	NR

**Table 5.** Data extraction—pesticides

Author/year	Country/income status	Study design	Study characteristics	Time of data collection (incl follow up)	Cognitive descriptions	Generation of DNA methylation	Environmental exposure	Type of association analysis	Covariates	Outcome: main analysis	Outcome: sex
Kimberly Paul/2018 [64]	USA (high)	Case–control	Sample size: 580 (PEG1)* (341 PEG1 PD cases, 238 PEG1 controls) Mean age: PEG1 (blood)—case mean 70.2 years, control mean 67.5 years Deprivation NR Ethnicity: PEG1: case 81% European ancestry, control 87% European ancestry Sex: PEG1: case 58% M, control 53% M *Study also reported findings from PEG2 saliva samples but these were not stratified by PD status and therefore cannot tell us about differential methylation per PD status	NR	Cases were enrolled within 3 years of PD diagnosis with confirmed idiopathic PD through in-person examinations at UCLA by a movement disorder specialist	Sample: peripheral whole blood DNAm: Illumina Infinium 450k platform *Study also reported findings from PEG2 saliva samples but these were not stratified by PD status and therefore cannot tell us about differential methylation per PD status	Specific exposure: Organophosphate pesticide Measurement: Occupational or residential exposure to 36 different organophosphate pesticides. Estimated from residential and occupational proximity to commercial agricultural pesticide applications through GIS model. Summed pounds of pesticide applied per year and per acre within a 500 metre buffer of each residential and occupational address of participants. Environmental context: PEG is based on participants from three agricultural counties in Central California Exposure time/length: Yearly average exposure over the study period (1974 to year of blood draw)	Chi squared or Pearson correlation for variation in demographics, cell composition and mean methylation by environmental exposure EWAS: regression of CpG methylation levels on confounders to form residuals. Correlation coefficients calculated for association testing implementing biweight mid-correlation calculation between residuals at each CpG and environmental exposure (p values based on Students *t*-test). Adjustment for multiple testing: Bonferroni correction (*P*-value<.05/500 000 = 10^e-08^) for genome-wide significance, and *P*-value<10^e-07^ for suggestive associations (FDR<7^e-03^). Analyses were conducted in the full population and stratified by PD status. Genome-wide analysis: PEG1 blood-based DNAm markers and analysed the CpGs with *P*-value<10^e-07^ in the PEG2 saliva-based DNA methylation.	Age, sex, PD, European ancestry Secondary analysis adjusted for cell count	Outcome of interest: Lower global mean methylation associated with higher pesticide exposure in blood and saliva samples. Pearson correlation: *r* = −0.13, *P* = .021 (blood), *r* = −0.18, *P* = .068 (saliva). 7 CpGs specific to cases: *MYH15* (cg03329597) *MFAP2* (cg15600437) *KIAA0319* (cg18433519) Other PD-specific CpGs intergenic or in genes of unknown function (LOC283267). Other outcomes: 27 CpGs identified at Bonferroni adjusted significance level related to organophosphate exposure: Top hit: *ALOX12* (cg01600516) Cases: *r* = 0.29, *P* = 4.11^e-08^ Controls: *r* = 0.21, *P* = 1.04^e-03^ Other top hits: *POLR1B* (cg15177604): *r* = −0.27, *P* = 7.70^e-11^ Intergenic (cg03655023): *r* = 0.27, *P* = 8.3^e-11^ *BRDT* (cg01081438): *r* = −0.26, *P* = 4.74^e-10^ *BANF2* (cg24859648): *r* = −0.26, *P* = 5.70^e-10^ Additional 43 CpGs associated with pesticide exposure at lower significance (*P* < 10^e-07^, FDR < 6.88^e-03^).	NR
Rodney Go/2020 [65]	Hawaii, USA (high)	Cross-sectional	Sample size: Stratified by years of work: 13 with 0 years, 4 with ≥10 years Stratified by number of organochlorines (OGCs) detected: 12 with 0–2, 4 with +4 detected (Recruited from the Honolulu Heart program) Mean age, stratified by years of work: 0 years mean age of death 88.8 years ≥10 years mean age of death 85.1 years Deprivation: Stratified by years of work: 0 years—mean years of education 11.8 ≥10 years—mean years of education 8 Ethnicity NR Sex: 100%M	Original cohort was men born 1900–19	All participant’s had PD diagnoses confirmed by a neurologist before death then confirmed Lewy Bodies in post-mortem brain samples, last CASI score before death and BRAAK_PD score based on immunohistochemical stain	Sample: peripheral blood (collected antemortem) DNA isolation: PureGene system (Gentra Systems, Inc.) DNA quantification: PicoGreen staining (Molecular Probes) DNA extraction from frozen brain sample: AllPrep DNA/RNA kit (Qiagen, Inc) Bisulfite treatment: EZ-96 DNA methylation kit (Zymo Research) DNAm: Illumina Infinium methylation assay and the Human Methylation450K BeadChip	Specific exposure: Occupational exposure to OGCs Measurement: Self-reported years of plantation work and organochlorine presence in brain tissue (occipital samples). Concentration was reported in parts per billion for 21 OGCs—they were considered present in the tissue if they were detected at more than the level of calibration Environmental context: All participants lived in Oahu, Hawaii during recruitment Exposure time/length: Self-reported years of work	Multivariate analysis of co-variance (ANCOVA) to assess the contribution of specific factors to the differences in methylation, including factors such as tissue type (brain vs. blood), OGC levels, plantation work exposure, age at blood draw (for blood), age at death (autopsy) and time to autopsy (post- mortem interval, for brain tissue). Principal component analysis of the 20 blood and brain tissue samples	Blood—age at blood draw Brain—autopsy (age at death), time to autopsy (post-mortem interval), and their interaction	Outcome of interest: Brain: 94 DMLs in postmortem brain tissue for plantation work exposure (≥10 years vs. 0 years), *P* < .001. Top hits: *PTGDS* gene: *P* = 3.28^e-06^ *PEX19* gene: *P* = 4.98^e-05^ Blood: Plantation work exposure (10+ years vs. 0 years): 788 DMLs, *P* < .001 123 DMLs, *P* < .0001 Top hits: *WNT16* gene: *P* = 9.04^e-07^ *ENTPD8* gene: *P* = 1.47^e-06^ Brain OGC Analysis: 69 DMLs, *P* < .001 8 DMLs, *P* < .0001 Top hit: *PACS2* gene (two loci): *P* = 1.18^e-06^, *P* = 1.60^e-05^ Blood OGC Analysis: 176 DMLs, *P *< .001 18 DMLs, *P* < .0001 Top hits: *DNAJC15* gene: *P* = 9.55^e-06^ *AP2A2* gene: *P* = 1.10^e-05^ Other outcomes: PCA showed distinct separation between high vs. low exposure and 4+ OGCs vs. 0–2 OGCs. Concordance analysis: 2 hypermethylated DMLs for *DNAJC15* in brain and blood between OGC exposure groups. 6 concordant genes associated with plantation work or OGC exposure in blood and brain. Validation: 6/7 DMLs validated in brain using pyrosequencing. 6/8 DMLs validated in blood using pyrosequencing.	NA
William Casazza/2023 [66]	France (high)	Case–control	Sample size: 71 early-stage PD, 148 matched (on age, sex, and region of residence) controls. Mean age: Case and control mean age 67 years. Deprivation NR. Ethnicity NR. Sex: Case 54%M, Control 55%M	NR	PD diagnosed according to UK Parkinson’s Disease Society Brain Bank criteria by a neurologist specialising in movement disorders. PD was defined as the presence of parkinsonism with exclusion of drug-induced phenotypes or further nervous system involvement. MMSE	Sample: whole-blood DNAm: Illumina HumanMethylationEPIC BeadChip array	Specific exposure: Occupational or domestic pesticide exposure Measurement: Pesticide exposure from self-reported questionnaire. Those that responded yes to having exposure: occupational health interviews were conducted to obtain detailed information—number and type of farms where the individuals had worked, which pesticides they had personally sprayed, the frequency, duration, and method of spraying, and number of years that they were exposed Environmental context: Discovery cohort TERRE consisted of agriculture workers living in France Replication cohort DIG-PD consisted of people living in France of similar demographics (sex, age) but this cohort had missing data for 88.6% participants in terms of pesticide exposure (117 cases, 112 controls) Exposure time/length: Self-reported years of exposure	PD genetic risk score calculated using summary statistics from a publicly available GWAS meta-analysis of PD case/control. EWAS for association between PD genetic risk score and DNAm. Cell type proportion analysis Mendelian Randomization to assess if colocalized mQTLs showed evidence of a causal association between DNAm and PD	Genetic risk score and EWAS—sex, age, genotyping principal components [1–3], DNAm principal components [1–10]. Associations tested for robustness of sex, age, smoking, alcohol, prior head injury, GRS.	Positive trend in predicted PD risk with increasing GRS quartiles: Fourth quartile: OR = 2.60; 95% CI = [1.03, 6.55]; *P* < .042 Replication cohort showed similar trend: Fourth quartile: OR = 2.84; 95% CI = [1.33, 6.09]; *P* < .0072 Pesticide exposure had little to no effect on DNAm when accounting for GRS. Nongenetic risk factors did not moderate the GRS-DNAm relationship. No other analyses reported on pesticide exposure in relation to DNAm sites and PD.	Not pesticide related—When stratifying GRS analysis by sex, GRSpT = 5^e-8^ uniquely associated with DNAm at five CpG sites in males and uniquely associated with DNAm at three CpG sites in females.
Karen Huen/2018 [54]	USA (high)	Cohort	Sample size: 238 mother/infant pair (185 with methylation data at two timepoints—newborn and 7 years) (from the CHAMACOS study) Mean age: mother’s age at enrolment mean 25.7 years Child age at 7 years assessment mean 7.1 years Deprivation measure: Poverty level during pregnancy—60% below poverty level Ethnicity NR Sex: Children 48%M 52%F	1999–2000 cohort enrolment	Child cognition at aged 7 years with WISC Fourth Edition (WISC-IV)	Sample: umbilical cord blood (after delivery), whole blood from children at 7 years DNA isolation: QIAamp Blood DNA Maxi kit (Qiagen, Inc., Santa Clarita, CA, USA Bisulfite conversion: Zymo Bisulfite conversion Kits (Zymo Research, Orange, CA, USA) DNAm: Illumina Infinium 450 K DNA methylation BeadChip	Specific exposure: Organophosphate pesticides—three dimethyl (DM) phosphate metabolites (dimethylphosphate, dimethylthiophosphate, dimethyldithiophosphate) and three diethyl (DE) phosphate metabolites (diethylphosphate, diethylthiophosphate, and diethyldithiophosphate) Measurement: DAP metabolites in maternal urine at 13- and 26-week gestation—proxy of organophosphate pesticide exposure. Maternal urine analysis with isotope dilution calibration Urine collected week 13 and week 26 gestation Environmental context: Mothers recruited from the agricultural region of Salinas Valley, California Exposure time/length: NR	Linear regression for the relationship of *PON1* DNAm at birth and 7 years with cognition. Linear regression of mean OP metabolites with DNAm at *PON1* CpGs. Mediation analysis with PARAMED module for CpGs with suggestive association with metabolites and cognitive outcomes. Secondary analysis—sex stratification, adjustment for child *PON1* genotype, adjustment for maternal *PON1* genotype Cell-type proportions were calculated. For cord blood samples, they used a cord blood reference dataset that included nucleated red blood cells. For blood samples from seven-year-old-children, they used an adult reference.	Maternal education, maternal verbal cognition scores, batch, and cell composition	Outcome of Interest: No CpGs significantly associated with Full Scale IQ test at 7 years after controlling for FDR Other Outcomes (Found in supplementary material): CpG site 3 and 7 associated with DE phosphates: CpG site 3: −0.08 (95% CI: −0.14, −0.01), *P* = .02 CpG site 7: −0.07 (95% CI: −0.12, −0.01), *P* = .03 Mediation analysis showed: Direct effect of prenatal diethyl DAPs on verbal comprehension IQ: Controlled Direct Effect estimate (95% CI): −5.2 (−10.1, −0.2) No significant effect on DNAm. Significant indirect effect of 7-year-old blood methylation at CpG site 1: Estimate (95% CI): −0.42 (−1.77, −0.002)	Sex stratification did not significantly change results

**Table 6.** Data extraction—pathway extraction

Author/year	Environmental exposure	Cognitive outcome	Information from formal analysis by authors
Air pollution			
Zhenjiang *et al*. [2023, 55]	Traffic-related PM2.5	Braak stage, CERAD and ABC score	Gene ontology analysis of top 1000 CpGs with lowest raw *P*-values for the PM2.5 EWAS and neuropathic markers. No pathways reached significance after correcting for multiple tests. Of the top 10 pathways for each of the PM2.5 exposures and neuropathic markers, one pathway was associated with both 3-year exposure to PM2.5 and CERAD. Eight genes (*HSPA1A*, *HSPA1L*, *IRS1*, *KRAS*, *NRAS*, *RPTOR*, *IRS2*, *ATG5*) within this pathway were enriched by differentially methylated CpG sites which were associated with 3-year PM2.5 exposure, and 10 genes (*ADCY3*, *ADCY5*, *NFKB1*, *PRKAG2*, *RPTOR*, *TSC2*, *EHMT1*, *ULK1*, *AKT1S1*, *ATG5*) with CERAD score.
Guo *et al*. [2022, 56]	Air pollution PM2.5 and NO_2_ Additionally analyzed chemicals—BPA, CPF, the sum of phthalate DEHP metabolites, PBDEs, PAH, PAH–DNA adducts	FSIQ of WISC-IV	For each neurodevelopmental outcome, keywords were defined and related biological pathways identified via the KEGG database No pathways were identified for IQ that were significantly associated
Lee *et al*. [2017, 53]	PAH from a coal power plant	WISC	No formal analysis
Proximity to roads			
Peng *et al*. [2018, 57]	Traffic-related pollution from living in proximity to major roads	KBIT-2, WRAML2 and WRAVMA	No formal analysis
Heavy metals			
Nielsen *et al*. [2015, 58]	Heavy metals such as manganese from welding fumes	Parkinsonism from UPDRS3 score	No formal analysis
Yang *et al*. [2015, 59]	Aluminum from occupational exposure	MCI, MMSE	No formal analysis
Castillo *et al*. [2017, 60]	Heavy metal exposure from mining activity	Parkinson’s disease	No formal analysis
Paul *et al*. [2021, 61]	Lead	Parkinson’s disease	No formal analysis
Cardenas *et al*. [2017, 62]	Mercury	PPVT and WRAVMA	No formal analysis
Wan *et al*. [2021, 63]	Heavy metals such as lead from living in proximity to a steel mill	CRT-C2	Function enrichment analysis Using WebGeStalt pathway database: DMRs involved in melanogenesis pathway (*P* = .03) Using WebGeStalt for gene ontology analysis: no significant enrichments Using Enrichr MGI Mammalian Phenotype 2017 database: DMRs involved in calcium phosphate metabolism (*P* = .019), elevated circulating parathyroid hormone levels (*P* = .019), hyperphosphataemia (*P* = .019), and retinitis pigmentosa (*P* = .019) Using WebGeStalt for disease analysis: DMRs associated with pseudohypoparathyroidism (PHP), unconsciousness, and pseudopseudohypoparathyroidism (PPHP) (*P* = .001 for unconsciousness; *P* < .001 for PHP and PPHP)
Pesticides			
Paul *et al*. [2018, 64]	Organophosphate pesticides	Parkinson’s disease	Formal analysis which provides insights of how pesticide exposure impacts gene expression (not specific to neurodegenerative disease or cognition): PANTHER gene ontology classification on genes with significant differentially methylated CpGs to predict function- One pathway identified with near significant overrepresentation after multiple testing correction—nicotinic acetylcholine receptor signaling pathway fold enrichment of 15.63 (Fisher’s exact *P*-value = 1.01^e-03^, FDR = 1.64^e-01^). Of top 1077 CpGs identified in the study mapped to 662 genes, the one significant overrepresented pathway was muscarinic acetylcholine receptor 1 and 3 signaling pathway fold enrichment of 3.90 (Fisher’s exact *P*-value = 5.36^e-04^, FDR = 4.73^e-02^). Both acetylcholine pathways were among the top pathways identified when only using data from controls. Other enrichments were located within the 662 genes such as pyrophosphatase-related catalytic activity (*P*-value = 2.75^e-05^, FDR = 2.64^e-03^) and postsynaptic membrane components (*P*-value = 1.33^e-03^, FDR = 2.13^e-02^).
Casazza *et al*. [2023, 66]	Organochlorine pesticides	Parkinson’s disease, MMSE	No formal analysis
Go *et al*. [2020, 65]	Pesticide	Parkinson’s disease, CASI, Braak score	Ingenuity pathway analysis Comparing ≥10 years plantation work to 0 years, mapping identified DML to genes and pathways: Top brain pathways—revealed “neurological disease; cell development, survival and death; and nervous system development and function” Top blood pathways—“neurological disorders,” e.g. movement disorders, neuromuscular disease, and Parkinson’s disease The most important genes among 15 genes with DML in these pathways: Androgen Induced 1 (*AIG1*), glutamate ionotropic receptor NMDA type subunit 2A (*GRIN2A*), B-Cell CLL/Lymphoma 2 (*BCL2*), Serum/Glucocorticoid Regulated Kinase 1 (*SGK1*), and microtubule associated protein gene (*MAPT*). Comparing 4+ OGCs to 0–2 OGCs detected, mapping DML to genes and pathways: Top brain and blood pathways: related neurological disease, inflammatory response, and nervous system development. When comparing high and low exposure to OGCs, 27 genes with blood DML were found in pathways related to mitochondrial and neuronal function, including genes such as Potassium Calcium-Activated Channel Subfamily N Member 3 (*KCNN3*), Microtubule Associated Protein 1B gene (*MAP1B*) and EPH Receptor A4 (*EPHA4*)
Huen *et al*. [2018, 54]	Organophosphate pesticides	WISC-IV	No formal analysis

### Risk of bias assessment

Risk of bias assessment was carried out by two independent reviewers (S.G. and C.H./J.I.). Any disagreements were resolved with the aid of third reviewer (AJM/RH). Included cross-sectional studies were assessed using the BIOCROSS evaluation tool [67]. BIOCROSS uses five domains relating to study design, methodology, analysis, interpretation, and biomarker measurement to evaluate the quality of biomarker-based studies. Cohort studies were assessed by a separate tool, the Joanna Briggs Institute Critical Appraisal Tool for Cohort Studies [68] (Available from: https://jbi.global/critical-appraisal-tools). Preprint articles were assessed through the AACODS (Authority, Accuracy, Coverage, Objectivity, Date, Significance) checklist [69] (Available from: https://dspace.flinders.edu.au/jspui/bitstream/2328/3326/4/AACODS_Checklist.pdf). Risk of bias assessment for all studies can be found in Supplementary Files Section 3. No study was excluded based on their risk of bias assessment.

### Evidence synthesis

Studies were assessed to determine if they had identified statistically significant associations between an environmental exposure and cognitive function, cognitive impairment, or neurodegenerative disease through DNAm. Statistical significance was equated to individual study standards outlined in Tables 2**–**6. Statistically significant DNAm sites were extracted along with their gene association and developed into a table (Table 7) for ease of comparison. Additionally, identified significant or top hit biological pathways from within-study analysis were extracted and developed into a table (Table 8) for ease of comparison.

**Table 7.** Identified CpGs and global methylation

Author/year	Cognitive outcome	Environmental exposure	Epigenetic association	Gene association
Air pollution				
Zhenjiang *et al*. [2023, 55]	Overlapping CpG from EWAS (1–4) (1) CERAD (2) Braak stage (3) CERAD (4) Braak stage and ABC score	Overlapping CpG from EWAS (1) 1- and 3-year PM2.5 (2) All PM2.5 windows (3) 1-year PM2.5 (4) 3- and 5-year PM2.5	[1] cg01835635 [2] cg09830308 [3] cg16342341 [4] cg27459981	[1] *APOA4* [2] *MLKL* [3] *SORBS2* [4] *MLKL*
Guo *et al*. [2022, 56]	FSIQ of the WISC-IV	Air pollutants PM2.5 and NO_2_ Also PAH and other chemicals	No significant findings	NA
Lee *et al*. [2017, 53]	WISC	PAH from a coal power plant	No significant findings	NA
Proximity to roads				
Peng *et al*. [2018, 57]	KBIT-2 nonverbal score (1–4)	Traffic-related exposures from living in proximity to roadway	[1] Hypermethylation cg05654765 [2] Hypermethylation g14099457 [3] Hypermethylation cg03732535 [4] Hypermethylation cg02954987	[1–4] *LAMB2*
Heavy metals				
Nielsen *et al*. [2015, 58]	Parkinsonism (UPDRS3 ≥ 15) Parkinsonism (UPDRS3 ≥ 15) Lower prevalence of Parkinsonism	Heavy metal exposure (all welding workers, full sample) Heavy metal exposure (excluding retired welders) Heavy metal exposure (non-retired welders)	Mean *NOS2* hypomethylation CpG site 3 8329 hypomethylation CpG site 8329 hypermethylation	*NOS2* *NOS2* *NOS2*
Yang *et al*. [2015, 59]	MCI	Aluminum (serum)	Global hypomethylation	NA
Castillo *et al*. [2017, 60]	Parkinson’s disease	Exposed or not directly exposed to mining activity in a high mining activity city	Global hypomethylation	NA
Paul *et al*. [2021, 61]	Parkinson’s disease Parkinson’s disease	Cumulative lead Cumulative lead	Higher DNAm tibial- lead in SGPD and PEG Lower DNAm patella-lead in SGPD	NA NA
Cardenas *et al*. [2017, 62]	Male PPVT score (1–4)	Maternal blood mercury	[1] cg05342682 hypomethylation [2] cg21856205 hypomethylation [3] cg17330251 hypomethylation [4] cg01874867 hypomethylation	[1–4] *PON1*
Wan *et al*. [2021, 63]	CRT-C2	Blood lead levels	Hypomethylation Hypomethylation	*FAM50B1* *PTCHD3*
Pesticides				
Casazza *et al*. [2023, 66]	Parkinson’s disease	Pesticide	No significant findings	NA
Paul *et al*. [2018, 64]	Parkinson’s disease CpGs from EWAS found only in Parkinson’s disease patients (1–3)	Higher organophosphate exposure Organophosphate exposure	Global hypomethylation [1] cg03329597 [2] cg15600437 [3] cg18433519	NA [1] *MYH15* [2] *MFAP2* [3] *KIAA0319*
Go *et al*. [2020, 65]	Top 5 hits from brain tissue of Parkinson’s disease patients (1–5) Top 5 hits from blood samples of Parkinson’s disease patients (6–10) Top 5 hits from brain tissue of Parkinson’s disease patients (11–15) Top 5 hits from blood samples of Parkinson’s disease patients (16–20)	≥10 years vs. 0 years of plantation work ≥10 years vs. 0 years of plantation work 4+ vs. 0–2 organochlorine detection 4+ vs. 0–2 organochlorine detection	[1] Hypermethylation cg14608180 [2] Hypermethylation cg08142821 [3] Hypermethylation cg17880320 [4] Hypermethylation cg06173536 [5] Hypermethylation cg11706717 [6] Hypermethylation cg25608490 [7] Hypomethylation cg12179661 [8] Hypermethylation cg08435683 [9] Hypermethylation cg02286380 [10] Hypermethylation cg16395997 [11] Hypermethylation cg18397450 [12] Hypermethylation cg18912855 [13] Hypomethylation cg02101742 [14] Hypermethylation cg09677945 [15] Hypermethylation cg15988970 [16] Hypermethylation cg09677945 [17] Hypomethylation cg12456927 [18] Hypomethylation cg02598807 [19] Hypomethylation cg11667387 [20] Hypermethylation cg15723784	[1] *PTGDS* [2] Intergenic [3] *PEX19* [4] *GNG4* [5] *NID1* [6] *WNT16* [7] *ENTPD8* [8] *SLC23A2* [9] *ARRB2* [10] *WDR8* [11] *PACS2* [12] *PACS2* [13] *TFDP1* [14] *DNAJC15* [15] *DNAJC15* [16] *DNAJC15* [17] Intergenic [18] *AP2A2* [19] *CNNM2* [20] *SMAD3*
Huen *et al*. [2018, 54]	WISC-IV	Organophosphate pesticide	No significant findings	NA

Table showing top CpGs significantly associated with environmental exposure and cognitive outcome in each study. Results shown for global DNAm where individual CpGs were not examined. Note: studies generally identified further CpGs significant with the environmental exposure and cognitive outcome individually

**Table 8.** Identified biological pathways including genes involved

Author/year	Environmental exposure	Cognitive outcome	Pathways	Genes
Air pollution				
Zhenjiang *et al*. [2023, 55]	Traffic-related PM2.5	Braak stage, CERAD and ABC score		*HSPA1A* *HSPA1L* *IRS1* *KRAS* *NRAS,* *RPTOR* *IRS2* *ATG5* *ADCY3* *ADCY5* *NFKB1* *PRKAG2* *RPTOR* *TSC2* *EHMT1* *ULK1* *AKT1S1* *ATG5*
Guo *et al*. [2022, 56]	PM2.5 and NO_2_ Also chemicals BPA, CPF, the sum of phthalate DEHP metabolites, PBDEs, PAH, PAH–DNA adducts	FSIQ of WISC-IV	No significant pathways	
Lee *et al*. [2017, 53]	PAH from a coal power plant	WISC	NA	NA
Proximity to roads				
Peng *et al*. [2018, 57]	Traffic-related pollution from living in proximity to major roadways	KBIT-2, WRAML2 and WRAVMA	NA	NA
Heavy metals				
Nielsen *et al*. [2015, 58]	Heavy metals such as manganese from welding fumes	Parkinsonism from UPDRS3 score	NA	NA
Yang *et al*. [2015, 59]	Aluminum from occupational exposure	MCI, MMSE	NA	NA
Castillo *et al*. [2017, 60]	Heavy metal exposure from mining activity	Parkinson’s disease	NA	NA
Paul *et al*. [2021, 61]	Lead	Parkinson’s disease	NA	NA
Cardenas *et al*. [2017, 62]	Mercury	PPVT and WRAVMA	NA	NA
Wan *et al*. [2021, 63]	Heavy metals such as lead from living in proximity to a steel mill	CRT-C2	Melanogenesis pathway Calcium–phosphate metabolism Elevated circulating parathyroid hormone levels Hyperphosphataemia Retinitis pigmentosa PHP, unconsciousness, and PPHP	
Pesticides				
Paul *et al*. [2018, 64]	Organophosphate pesticides	Parkinson’s disease	Nicotinic acetylcholine receptor signaling pathway Muscarinic acetylcholine receptor 1 and 3 signaling pathway Pyrophosphatase related catalytic activity Postsynaptic membrane components	
Casazza *et al*. [2023, 66]	Pesticide	Parkinson’s disease, MMSE	NA	NA
Go *et al*. [2020, 65]	Organochlorine pesticides	Parkinson’s disease, CASI, Braak score	Neurological disease or disorders, e.g. movement disorders, neuromuscular disease and Parkinson’s disease Cell development, survival, and death Nervous system development and function Inflammatory response	*AIG1* *GRIN2A* *BCL2* *SGK1* *MAPT* *KCNN3* *MAP1B* *EPHA4*
Huen *et al*. [2018, 54]	Organophosphate pesticides and other chemicals	WISC-IV	NA	NA

After assessing the study outcomes and study methodologies, authors subjectively determined that heterogeneity would prevent the outcomes from studies being reliably combined for meta-analysis. We therefore opted to pursue a narrative synthesis which would allow us to produce a comprehensive summary of study findings.

## Results

Our search strategy yielded 6886 studies for title and abstract screening after the removal of duplicates. During this stage, we achieved an 87% agreement rate and selected 125 studies for full-text screening. After assessing these studies against our eligibility criteria (Table 1), we reached a 95% agreement rate and selected 14 studies to be included in our review. Studies included in this review were split according to the environmental exposures they investigated to provide us with a summary of the literature on specific exposures and enable us to easily identify knowledge gaps. Studies focused on air pollution (*n* = 3) [53, 55, 56], proximity to the roads (*n* = 1) [57], heavy metals (*n* = 6) [58–63], or pesticides (*n* = 4) [54, 64–66].

### Air pollution

#### Study characteristics

All studies took place in upper-middle (China *n* = 1 [53]) or high-income countries (USA, *n* = 2 [55, 56]). Zhenjiang *et al*. [55] had a cross-sectional design investigating adults of mean age 76.6 years, while Lee *et al*. [53] and Guo *et al*. [56] were cohort studies investigating mother/infant pairs. Children in the Lee *et al*.’s [53] study were ∼5 years during cognitive testing and children in Guo *et al*.’s [56] study were ∼5 and 7 years during cognitive testing. The mean sample size of the included studies was 268 (range 159–341). Participants in Zhenjiang *et al*.’s [55] study were majorly male (56%) while Lee *et al*.’s [53] study included mostly female participants (74% in 2002 cohort). Guo *et al*. [56] did not report the sex of included participants.

#### Environmental exposure

Zhenjiang *et al*. [55] investigated estimated traffic-related PM2.5 1, 3, and 5 years predeath by matching participants’ residential address to the closest calculated PM2.5 grid. Guo *et al*., [56] investigated exposure to chemicals including polycyclic aromatic hydrocarbons (PAH) through examining maternal urine during pregnancy. Guo *et al*. [56] additionally investigated PM2.5 and NO_2_ through identifying average daily measurements of these pollutants throughout pregnancy. Lee *et al*. [53] investigated PAH exposure from a coal plant through analyzing maternal (postpartum) and umbilical cord white blood cell DNA. Lee *et al*. [53] was interested in identifying the influence of a nearby coal plant, samples were therefore from participants in two different cohorts, one cohort’s samples were collected preclosure of the coal plant and the other cohort samples were collected postclosure.

#### DNAm

Zhenjiang *et al*. [55] used postmortem prefrontal cortex samples to analyze DNAm at single-site resolution while Guo *et al*. [56] used umbilical cord blood to analyze DNAm at single-site resolution. Lee *et al*. [53] specifically assessed LINE-1 methylation in umbilical cord blood.

#### Cognitive outcomes

Regarding cognitive outcomes, Zhenjiang *et al*. [55] assessed participants for AD-related pathology through Braak score, CERAD and ABC score (formed from assessing amyloid deposits, Braak score and CERAD criteria [70]). Guo *et al*. [56] utilized the FSIQ and WISC-IV cognitive tests when children were 5 and 7 years old and, Lee *et al*. [53] utilised the WISC when children were 5 years old.

#### Evidence synthesis—DNAm sites

Overall, *n* = 1/3 of the included studies identified that DNAm was associated with air pollution (PM2.5) and a cognitive outcome.

##### Studies of adults (>18 years of age)

Zhenjiang *et al*. [55] study included 159 participants living within Atlanta, Georgia, the majority of which had received an AD or other dementia subtype diagnosis. Zhenjiang found evidence that prefrontal cortex DNA was differentially methylated per exposure to traffic-related PM2.5. Four CpG sites were identified which were statistically significant for both their environmental exposure and cognitive measure. CERAD score, 1- and 3-year exposure to PM2.5 were associated with cg01835635 (*APOA4*), Braak stage and 1-, 3- and 5-year exposure to PM2.5 was associated with cg09830308 (*MLKL*), CERAD score and 1-year PM2.5 exposure was associated with cg16342341 (*SORBS2*) and Braak stage, ABC score, 3- and 5-year PM2.5 exposure were all associated with cg27459981 (*MLKL*).

##### Studies of children (<18 years of age)

Guo *et al*. [56] study investigated 341 mother/infant pairs, mothers being residents of the Northern Manhattan or Southern Bronx areas of USA at least 1 year prior to pregnancy. Guo *et al*. [56] investigated chemicals such as PAH and air pollutants PM2.5 and NO_2_ and measured children’s cognitive function at 5 and 7 years old with the FSIQ of the WISC-IV and did not identify any statistically significant CpG sites when analyzing umbilical cord blood samples. Lee *et al*. [53] investigated 150 mother/infant pairs living within 2.5 km to the Tongliang power plant, stated to be the principal source of air pollution within their residing city. Authors measured PAH exposure from living in proximity to this power plant and measured children’s cognitive function using the WISC and concluded that when analyzing umbilical cord blood, LINE1 methylation did not mediate the relationship between PAH–DNA adducts and IQ.

#### Evidence synthesis—biological pathways

Overall, *n* = 2/3 studies performed a type of pathway analysis to further explore the impact of environmentally influenced DNAm on cognitive outcomes.

Zhenjiang *et al*. [55] investigated traffic-related PM2.5 exposure with Braak stage, CERAD and ABC score. Zhenjiang *et al*. [55] performed gene ontology analysis of the top 1000 CpGs, which associated PM2.5 and neuropathic markers. However, no pathways reached significance after correcting for multiple tests. Of the top 10 pathways identified through the ontology analysis, one pathway was associated with 3-year exposure to PM2.5 and CERAD score. Eight genes in this pathway were enriched by differentially methylation CpG sites associated with PM2.5 exposure (*HSPA1A, HSPA1L*, *IRS1*, *KRAS*, *NRAS*, *RPTOR*, *IRS2*, *ATG5*) and 10 genes with CERAD score (*ADCY3*, *ADCY5*, *NFKB1*, *PRKAG2*, *RPTOR*, *TSC2*, *EHMT1*, *ULK1*, *AKT1S1*, *ATG5*).

Guo *et al*. [56] did not identify any statistically significant pathways using the KEGG database which associated chemical (PAH) or air pollutant (PM2.5, NO_2_) exposure to FSIQ in children and Lee *et al*. [53] did not perform pathway analysis.

### Proximity to roads

To ensure we did not assume the interpretation of study outcomes, we separated air pollution and proximity to roads in our evidence synthesis as Peng *et al*. [57] did not specify an interest a specific impact of living in proximity to the road.

#### Study characteristics

Peng *et al*. [57] was the only study to explore proximity to the road as an environment exposure [57]. This study took place in the USA and was of a cohort design investigating 482 mother/infant pairs. Majority of the children in Peng *et al*.’s [57] study were male (52%) and the age of children included within the study was not reported.

#### Environmental exposure, DNAm, and cognitive outcome

Peng *et al*. [57] investigated traffic-related exposures from examining residential proximity to major roads and analyzed umbilical cord blood for DNAm at single-site resolution. For cognitive testing, the study used the Kaufman Brief Intelligence Test (KBIT-2), Wide Range Assessment of Memory and Learning (WRAML2), and Wide Range Assessment of Visual and Motor Abilities (WRAVMA) during the mid-childhood visit.

#### Evidence synthesis—DNAm

Participants in Peng *et al*.’s [57] study were a part of Project VIVA and comprised mother/infant pairs living around eastern Massachusetts. Peng *et al*. [57] identified four CpGs, which associated living in proximity to major roads to lower KBIT-2 scores in children when analyzing 415 umbilical cord blood samples including hypermethylation of cg05654765 (average reduction of −2.74 points), cg14099457 (average reduction of −2.28 points), cg03732535 (average reduction of −10.89 points), and cg02954987 (average reduction of −1.45 points) (all associated with *LAMB2*).

#### Evidence synthesis—biological pathways

Peng *et al*. [57] did not perform pathway analysis.

### Heavy metals

#### Study characteristics

All studies took place in upper-middle (China *n* = 2 [59, 63]) or high-income countries (USA *n* = 3 [58, 61, 62], Chile *n* = 1 [60], or Australia/New Zealand *n* = 1 [61]). Four studies investigated adults (>18 years of age). Nielsen *et al*. [58] had a nested case–control design and Castillo *et al*. [60] and Paul *et al*., [61] were both case–control studies, while Yang *et al*. [59] had a cross-sectional design. The mean sample size of adult studies was 867 (range 201–2696) and mean age was 55.5 years (range 25–72 years). Nielsen *et al*. [58] and Yang *et al*. [59] both only included male participants and both Castillo *et al*. [60] and Paul *et al*.[61] had majorly male participants. The remaining two studies investigated children (<18 years of age). Cardenas et al., [62] study had a cohort design investigating 321 mother/infant pairs while Wan *et al*. [63] was a cross-sectional study investigating 333 children. Children in Cardenas *et al*.’s [62] study were mean age 3.4 years and 7.9 years during their childhood visits while children in Wan *et al*.’s [63] study were mean age 9.8 years. Participants in both Cardenas *et al*.’s [62] and Wan *et al*.’s [63] study were majorly female (50.1% and 52.7%, respectively).

#### Environmental exposure

Nielsen *et al*. [58] investigated self-reported exposure to welding fumes in current and retired workers of welding sites; fumes at sites were hypothesized to contain high concentrations of manganese for example. Yang *et al*. [59] investigated self-reported occupational exposure to aluminum and Castillo *et al*. [60] investigated self-reported exposure to mining activity including occupational exposure to metals (no specific metals stated). Paul *et al*. [61] investigated long-term exposure to lead. Regarding children-centered studies, Cardenas *et al*. [62] investigated mercury exposure through analyzing maternal blood samples during pregnancy using the Direct Mercury Analyzer 80 and Wan *et al*. [63] investigated arsenic, cadmium, mercury, and lead exposure from living in proximity to a steel mill in children living in a town containing a steel mill versus living in a town not containing a steel mill.

#### DNAm

Yang *et al*. [59] and Castillo *et al*. [60] analyzed adult blood samples for global methylation, while Nielsen *et al*. [58] specifically assessed *NOS2* methylation and Paul *et al*. [61] took a unique approach to their study, using adult blood samples to develop DNAm biomarkers (as seen in the Normative ageing study) for lead exposure. Cardenas *et al*. [62] analyzed umbilical cord blood and blood samples from children taken at mean 7 years of age single-site resolution. Wan *et al*. [63] also analyzed blood samples from children at single-site resolution.

#### Cognitive outcome

Nielsen *et al*. [58] assessed participants for PD with the Unified Parkinson’s Disease Rating Scale 3 (UPDRS3), while Castillo *et al*. [60] and Paul *et al*. [61] assessed participants for PD according to clinical criteria. Yang *et al*. [59] assessed clinically diagnosed mild cognitive impairment and performed the MMSE. Cardenas *et al*. [62] used the Peabody Picture Vocabulary Test (PPVT) and WRAVMA when children were on average 7.9 years old (early childhood visit). Wan *et al*. [63] used the Combined Raven’s Test for China (CRTC2).

#### Evidence synthesis—DNAm

All studies (*n* = 6/6) identified that DNAm was associated with heavy metals (lead *n* = 2, manganese *n* = 1, aluminum *n* = 1, general exposure (no specific metals stated) *n* = 1, mercury *n* = 1) and a cognitive outcome.

##### Studies of adults (>18 years of age)

Nielsen *et al*. [58] study analyzed adult blood samples of 201 men from Midwest USA who had worked in one of three welding sites hypothesized to have resulted in high levels of manganese exposure. Nielsen *et al*. [58] found that UPDRS3 scores ≥ 15 indicating Parkinsonism were associated with lower mean *NOS2* methylation for workers and retirees of welding sites. Nielsen *et al*. [58] also found that UPDRS3 scores ≥ 15 indicating Parkinsonism were associated with lower CpG site 3 8329 of *NOS2* in workers of welding sites (relating to heavy metal exposure). Nielsen *et al*. [58] additionally found that each 1% increase in CpG site 8329 methylation in nonretired workers was associated with a 33% lower prevalence of Parkinsonism (95% CI: 8–51%). Paul *et al*. [61] study used participant data from the SGPD (959 case, 930 control) study based in Australia and New Zealand, and the PEG study (569 case, 238 control) based on 3 agricultural counties in the USA. Paul *et al*. [61] identified that PD was associated with a blood-based DNAm biomarker for tibia-lead levels in both the SGPD and PEG cohorts. Paul *et al*. [61] also identified that PD was associated with a blood-based DNAm biomarker for patella-lead in the SGPD cohort (OR 0.7). Paul *et al*.’s [61] findings remained statistically significant with sex-stratification. Yang *et al*. [59] study included 366 male participants from the same region of Zunyi in China who had regular aluminum exposure (mean duration of exposure 21.2 years). Yang *et al*. [59] found global hypomethylation was associated with MCI (OR 3.66) and serum aluminum concentrations (OR 1.76) in their blood samples. Castillo *et al*.’s [60] study compared participants living in a high-mining activity city accounting for 51% of Chile’s mineral production (i.e. production of copper, lithium, natural nitrates, iodine, molybdenum, gold, silver, and borates) to participants living in a city with lower mining activity (0.7% of Chile’s mineral production). Castillo *et al*. [60] found global hypomethylation in blood samples of PD patients who were directly exposed to mining activity in a high-mining activity city compared to those who self-reported they had no direct exposure.

##### Studies of children (<18 years of age)

Cardenas *et al*.’s [62] study used participant data from 321 mother/infant pairs from Project VIVA. Cardenas *et al*. [62] identified four CpGs, which associated maternal blood mercury with male children’s PPVT scores in early childhood including hypomethylation of cg05342682, cg21856205, cg17330251, and cg01874867 (all associated with *PON1*). Wan *et al*. [63] included participants living within two neighbouring towns within the same city in China, one town being in proximity to an industrialized area with a steel mill. Wan *et al*. [63] identified two imprinted gene regions, which statistically mediated the relationship between children’s blood lead levels and IQ which included hypomethylation in *FAM50B1* and *PTCHD3*.

#### Evidence synthesis—biological pathways

One study (*n* = 1/6) performed a type of pathway analysis. Wan *et al*. [63] performed functional enrichment analysis, using numerous databases to identify pathways associated with identified DMRs associating heavy metal exposure such as lead from living in proximity to a steel mill and children’s cognitive function measured with the CRT-C2. Using the WebGeStalt pathway database, identified DMRs were involved in a melanogenesis pathway, while using WebGeStalt for gene ontology, no significant enrichments were identified. Using WebGeStalt for disease analysis identified DMRs were associated with PHP, unconsciousness and PPHP. When using the Enrichr MGI Mammalian Phenotype 2017 database, DMRs were identified to be involved in calcium–phosphate metabolism, elevated circulating parathyroid hormone levels, hyperphosphataemia, and retinitis pigmentosa.

### Pesticides

#### Study characteristics

All studies took place in high-income countries (USA *n* = 3/4 [54, 64, 65], France *n* = 1/4 [66]). Three studies investigated adults (*n* = 3/4). Paul *et al*. [64] and Casazza *et al*. [66] had a case–control design, while Go *et al*. [65] was a cross-sectional study. The mean sample size of adult studies was 277 (range 17–580) and the mean age of included participants was 74.3 years (range 67–88.8 years). Go *et al*. [65] only included male participants and Paul *et al*. [64] and Casazza *et al*. [66] studies had majority male participants (58% and 54.5%, respectively). The remaining study investigated children. Huen *et al*. [54] was a cohort study investigating 238 mother/infant pairs. Children were mean age 7.1 years during their cognitive assessment and 52% were female.

#### Environmental exposure

Paul *et al*. [64] investigated occupational or residential exposure to organophosphates, which was estimated from residential or occupational proximity to commercial agricultural applications and Go *et al*. [65] investigated self-reported occupational exposure to organochlorines (OGCs) including identifying OGCs in brain samples. The final adult study, Casazza *et al*. [66] investigated self-reported occupational or domestic pesticide exposure (type not specified). Huen *et al*. [54] child study investigated organophosphate pesticides, either diethyl or dimethyl phosphate metabolites through examining maternal urine samples during pregnancy with isotope dilution calibration.

#### DNAm

Paul *et al*. [64], Casazza *et al*. [66], and Go *et al*. [65] analyzed DNAm at single-site resolution using adult blood samples. Paul *et al*. [64] additionally analyzed global DNAm. Huen *et al*. [54], specifically assessed *PON1* methylation in umbilical cord blood and blood from childhood samples at mean age 7 years.

#### Cognitive outcome

Paul *et al*. [64] assessed participants for PD according to clinical criteria. Casazza *et al*. [66] assessed participants for PD according to clinical criteria and performed the MMSE. Go *et al*. [65] used postmortem samples of those with a PD diagnosis, these participants also had Cognitive Abilities Screening Instrument (CASI) and Braak score available from before death. Huen *et al*. [54] used the WISC-IV when children were 7 years as the cognitive measure.

#### Evidence synthesis—DNAm

Overall, *n* = 2/4 studies identified that DNAm was associated with pesticides (organophosphate *n* = 1, organochlorine *n* = 1) and a cognitive outcome, with both studies investigating adults.

##### Studies of adults (>18 years of age)

Paul *et al*. [64] used participant data from 590 individuals in the PEG study with blood samples and analyzed their occupational or residential exposure to 36 different organophosphate pesticides. Paul *et al*. [64] identified three CpGs which were only identified in PD patients as opposed to the controls which were associated with organophosphate exposure including cg03329597 (*MYH15*), cg15600437 (*MFAP2*) and cg18433519 (*KIAA0319*). Paul et al., [64] also identified that PD patient status was associated with higher organophosphate exposure and global hypomethylation. Go et al., [65] investigated 17 males who had lived in Oahu, Hawaii. Go et al., [65] identified many antemortem blood-derived DML which associated plantation work or organochlorine pesticide exposure with PD. The top five hits when comparing 10+ years of plantation work to 0 years of plantation work in post-mortem brain samples of PD patients included hypermethylation of cg14608180 (*PTGDS*), cg08142821 (Intergenic), cg17880320 (*PEX19*), cg06173536 (*GNG4*), and cg11706717 (*NID1*). When comparing plantation work in blood samples of PD patients, the top 5 hits included hypermethylation of cg25608490 (*WNT16*), cg08435683 (*SLC23A2*), cg02286380 (ARRB2) and cg16395997 (*WDR8*), and hypomethylation of cg12179661 (*ENTPD8*). When comparing PD patients with 4+ to 0–2 detected organochlorides in post-mortem brain samples, the top five hits included hypermethylation of cg18397450 (*PACS2*), cg18912855 (*PACS2*), cg09677945 (*DNAJC15*) and cg1598870 (*DNAJC15*) and hypomethylation of cg02101742 (*TFDP1*). When comparing 4+ to 0-2 organochlroides detected in PD patient’s blood samples, the top 5 hits included hypermethylation of cg09677945 (*DNAJC15*) and cg15723784 (*SMAD3*) and hypomethylation cg12456927 (Intergenic), cg02598807 (*AP2A2*) and cg11667387 (*CNNM2*). The concordant nature of the *DNAJC15* related CpGs should be noted here.

Casazza *et al*. [66] study included participants (88 early-stage PD cases, 177 controls) from the TERRE study, comprised of agricultural workers from France. Casazza *et al*. [66] concluded that there was no statistically significant impact of pesticides on blood-derived DNAm in PD patients when accounting for their PD genetic risk score.

##### Studies of children (<18 years of age)

Huen *et al*.’s [54] study investigated 238 mother/infant pairs (mothers from an agricultural region of Salinas Valley, California). Huen *et al*. [54] found no evidence that organophosphate pesticide exposure and measured children’s cognitive function at 7 years old using the WISC-IV impacted blood-derived methylation profiles of children.

#### Evidence synthesis—biological pathways

Two studies (*n* = 2/4) performed a type of pathway analysis, with both being adult studies.

Paul *et al*. [64] performed PANTHER gene ontology, which did not specifically look at the impact of environmental exposure on the cognitive outcome; however, it does provide us some insights regarding how pesticide exposure may impact gene expression. The nicotinic acetylcholine receptor signaling pathway and the muscarinic acetylcholine receptor 1 and 3 signaling pathway were found to be overrepresented. Pyrophosphatase-related catalytic activity and postsynaptic membrane components were also implicated within enriched genes.

Go *et al*. [65] investigated the relationship between pesticide exposure and PD and performed ingenuity pathway analysis. Top brain pathways identified from DMLs when comparing ≥10 years plantation work to 0 years included “neurological disease; cell development, survival and death; and nervous system development and function’ and top blood pathways included ‘neurological disorders,” e.g. movement disorders, neuromuscular disease, and Parkinson’s disease. The most important genes in these pathways were *AIGI*, *GRIN2A*, *BCL2*, *SGKI*, and *MAPT*. Top brain and blood pathways identified from DMLs when comparing 4+ detected organochlorines to 0–2 related to neurological disease, inflammatory response, and nervous system development. Additionally, when comparing high to low exposure to organochlorines, genes within DML identified in blood were located in pathways relating to mitochondrial and neuronal function, including genes such as *KCNN3*, *MAP1B*, and *EPHA4*.

### Risk of Bias Assessment

We performed risk of bias assessments (Supplementary File Section 3) on all included studies with different tools based on study design and according to if a study was a preprint article.

#### Cross-sectional studies

Cross-sectional studies (*n* = 7/14) [58–61, 63–65] studies were assessed with the BIOCROSS evaluation tool that assessed studies according to five domains—rationale, study design/methods, data analysis, data interpretation, and biomarker measurements. All studies consistently provided a suitable rationale, including study aims and objectives, as well as detailed methodologies. All studies performed particularly well in the data analysis domain centered around detailing statistical analysis. However, studies consistently performed poorly in the data interpretation domain in terms of failing to acknowledge the limitations of cross-sectional study including issues of causal interpretation. Studies also consistently performed poorly in the biomarker measurement domain, as studies did not discuss laboratory measurement issues, blinding, and standardization.

#### Cohort studies

Cohort studies (*n* = 5/14) [53, 54, 56, 57, 62] were assessed with the Joanna Briggs Cohort checklist that is comprised of 11 questions, which enable us to determine if studies adequately measured their exposures and outcomes and identified and handled confounding factors for example. All studies measured their exposures in a reliable way in all participants, identified and adjusted for confounding factors and provided explanations of their statistical techniques. However, no study provided information on whether participants were free of their outcomes (i.e. cognitive deficits) at the start of the study or before exposure, considering that the biological samples used in these studies were commonly umbilical cord blood, and the children underwent cognitive assessments at mean age of 5 and/or 7 years. Numerous studies also did not discuss reasons for follow-up loss which would have been desirable.

#### Preprint articles

Finally, we included two preprint articles [55, 66] in our review, which were assessed using the AACODS checklist. Zhenjiang *et al*.’s [55] study is of credible authorship and a clear aim, employing valid methodology, contributes valuable insights into PM2.5-associated DNAm in AD and underscores the need for further research in this relatively small field. Casazza *et al*.’s [66] study has clearly outlined objectives, and a comprehensive methodology to investigate the genetic and epigenetic factors influencing cognitive outcomes in PD among agricultural workers. This study offers valuable insights with additional causal investigation through Mendelian randomization.

## Discussion

There is currently a knowledge gap regarding the association of environmental exposures with the underlying biology behind cognitive impairment and neurodegenerative disease [12]. We systematically reviewed the literature on a range of environment exposures, DNAm and cognitive outcomes to identify possible statistically significant DNAm sites within this relationship and identify any evidence of biological pathways. Overall, 62.5% (*n* = 10/14) studies included in this review found evidence that certain environmental exposures were associated with cognitive function, cognitive impairment or neurodegenerative disease through either identifying CpG sites or looking at global DNAm (*n* = 1/3 air pollution (PM2.5), *n* = 1/1 proximity to roads, *n* = 6/6 heavy metals (lead, aluminum, manganese, mercury, and general metal exposure), *n* = 2/4 pesticides (organophosphate and organochlorine). None of the identified CpGs overlapped between studies as seen in Table 7. Five of these studies (*n* = 2 air pollution, *n* = 1 heavy metals, *n* = 2 pesticides) also investigated biological pathways. However, we did not identify any studies in the literature meeting our eligibility criteria which investigated light pollution, noise pollution, green or blue space, walkability, cyclability, or novel exposures such as microplastics.

### Air pollution

We identified three studies investigating the effects of air pollution on the DNAm profiles of adults relating to cognitive impairment or neurodegenerative disease (*n* = 1/3) or children relating to cognitive function (*n* = 2/3). Zhenjiang *et al*.’s [55] study was the only study to identify statistically significant associations. Zhenjiang *et al*. [55] assessed prefrontal cortex DNAm majorly in AD patients (95.6% AD or other dementia subtype diagnosis) and associated this with PM2.5 exposure. Four CpG sites were identified as being exposed with both a cognitive variable and PM2.5 exposure, two of these CpGs (cg09830308 and cg27459981) associated with the *MLKL* gene. *MLKL* is implicated in neuronal loss in AD by a process called necroptosis (recent summary review on this topic here [71]. *MLKL* is suspected to contribute to a molecular pathway involving both *RIPK1* and *RIPK3* [71–73]. Literature has associated air pollution to both *MLKL* and *RIPK* genes in human alveolar epithelial cells [74] and the ovarian follicles of mice [75]. It would be of interest for future work to validate the impact of air pollutants on this necroptosis pathway in human populations.

Lee et al., [53] study investigated the impact of PAH exposure from living in proximity to a coal plant with LINE1 methylation profiles and associating this with children’s WISC scores at 5 years of age. Lee et al., [53] did identify statistically significant associations between prenatal PAH exposure and LINE1 methylation and LINE1 methylation and cognitive scores individually however, LINE1 methylation did not significantly mediate the relationship between PAH-DNA adducts and cognitive scores. LINE1 methylation is associated with maintaining genomic stability [76] and has been implicated in studies of neurodegeneration, increasing LINE1 methylation being associated with AD [77]. A further study of late-onset AD patients did not find a statistically significant association between LINE1 methylation and blood samples of AD patients or controls [78] suggesting further work is required to determine the potential impact of LINE1 methylation on cognitive outcomes.

### Proximity to roads

Peng *et al*.’s [57] study was the only study to use proximity to road as their environmental exposure, potentially looking at the dual impact of air and noise pollution. Peng *et al*. [57] identified four hypermethylated sites in the *LAMB2* gene associated with lower cognitive scores in KBIT-2; however, these sites did not replicate in the Generation R study population. *LAMB2* mutations are implicated in Pierson syndrome [79], a rare disorder commonly involving neurodevelopmental impacts [80]. Peng *et al*. [57] performed additional interesting analysis, examining identified *LAMB2*-related sites in premortem blood samples and post-mortem brain samples of adults. Methylation levels were higher and more variable in blood samples compared to brain samples, suggesting methylation levels could be tissue specific. Further investigation of *LAMB2*-related sites could enable comparisons to be drawn in terms of differential epigenetic regulation of central and peripheral systems relating to cognition.

It is important to note and consider the potential overlap between studies investigating proximity to road and air pollution. In Zhenjiang *et al*.’s [55] study of air pollution included within this review, stated that they were interested in traffic-related PM2.5 exposure. Some data used within studies investigating air pollution is commonly modeled from road and traffic monitoring data. Zhenjiang *et al*.’s [55] study for example used road inventory and traffic monitoring data shared by the Georgia Department of Transportation. Given the potential overlap in data between studies of air pollution and proximity to roads, care should be taken to disentangle the influence of traffic-related exposures such as noise pollution and air pollution.

### Heavy metals

We identified six studies that investigated heavy metals, DNAm, and a cognitive outcome. Numerous included adult studies were interested in PD. Nielsen *et al*. [58], e.g., investigated *NOS*2 methylation levels in workers and retirees of welding sites, hypothesized to be exposed to heavy metals such as manganese. Nielsen *et al*. [58] found that UPDRS3 scores ≥ 15 indicating Parkinsonism was associated with mean *NOS2* hypomethylation in both workers and retirees, and *NOS2* hypomethylation at CpG site 3 8329 in workers. The hypomethylation found is contradictory to a study by Kile *et al*. [81] in which *NOS2*/iNOS methylation was significantly increased as years working as a welder increased (not relating to Parkinsonism) [81]. Nielsen *et al*. [58] did, however, suggest that considering promoter methylation of *NOS2* is associated with greater *NOS2*expression [82] and greater *NOS2*/iNOS activity may contribute to a nitric oxide pathway. Future study measuring *NOS2* methylation in addition to *NOS2* expression levels, *NOS2*/iNOS activity and nitric oxide levels could provide further insights on this hypothetical pathway.

Wan *et al*.’s [63] study was the only study of children <18 years of age included in our review, which did not assess mother/infant pairs. Wan *et al*. [63] investigated the influence of living near a steel mill (relating to heavy metal exposure) on children’s blood lead levels, methylation profiles, and cognitive scores. Hypomethylation of *FAM50B1* and *PTCHD3* gene regions was found to mediate the relationship between blood lead levels and children’s IQ measured with the CRT-C2. These gene regions were identified as imprinted genes within this study, suggesting further work on imprinted vs. nonimprinted gene susceptibility to impacts from environmental exposures could provide us with interesting/novel findings. Wan *et al*. [63] were additionally involved in a previous study which assessed the influence of cadmium, arsenic, and mercury co-exposure on children’s IQ using the CRT-C2 [83]. Children living in an industrial area with more direct exposure to heavy metals (living in a town with smelter and steel plants) had statistically significant lower IQ scores to children without direct exposure to metals (living in a town in the same district without smelter or steel mills) (*P* < .05). In both Wan *et al*.’s [63] and Pan *et al*.’s [83] studies, blood lead levels were associated with decreased IQ (*P* < .001). Lead has been associated with lower cognitive function in other studies [84] and is also implicated in AD risk [85, 86]. These studies showcase that the impact of heavy metal exposure is being investigated with regard to cognitive outcomes. Where possible, exploring differential methylation profiles and further co-exposure investigation in future heavy metal work will aid in advancing the field.

Other studies in the literature have been investigating differential DNAm profiles in adults in response to heavy metals such as Freydenzon *et al*. [87], investigating heavy metal exposure and DNAm in an amyotrophic lateral sclerosis (ALS) case/control group [87]. Freydenzon *et al*. [87] identified cadmium exposure was associated with hypermethylation of cg03085637 (in proximity to *GNRHR2* and within *PEX11B*) and cg16655883 (*ZFR2*), and hypomethylation of cg21124714 (*P2R76*). In addition, hypermethylation of cg07503918 (*PRKG1-AS1*) was associated with metallurgy while mercury exposure replicated a similar effect for cg03085637 as cadmium exposure and was associated with hypomethylation of cg16845679 (an intergenic CpG site on chromosome 12) (all associations considered statistically significant at *P* < 3.19^e-7^) [87]. ALS was included as a covariate in this study as the study focused on identifying associations between heavy metal exposure and DNAm, therefore was not included in our evidence synthesis according to our eligibility criteria.

### Pesticides

We identified four studies which investigated pesticide exposure. Casazza *et al*. [66] were interested in PD, assessing if PD genetic risk score correlated with DNAm profiles. In their main analysis, Casazza *et al*. [66] found that pesticide exposure (no specific type stated) had no statistically significant impact on DNAm in PD patients when accounting for their genetic risk score. Subsequent Mendelian randomization analysis not relating to pesticide exposure was performed to assess if colocalized mQTLs (genetic variants within a similar or the same gene region) identified within the study, were causally associated with levels of DNAm and PD [66]. The largest causal effects identified were on chromosome 17, including cg19276540 (*MAPT*) where in hypermethylation coincided with decreased PD risk. Additionally, cg10673740 (*BAG3*) hypermethylation coincided with decreased PD risk. MAPT encodes the tau protein which is directly related to tauopathy. Tau hyperphosphorylation is associated with AD, Pick’s disease, frontotemporal dementia and progressive supranuclear palsy [88, 89]. *MAPT* mutations have also been associated with frontotemporal dementia with Parkinsonism [90, 91]. Additionally, *BAG3* is suspected to contribute to tau clearance [92]. It would be of interest to replicate these findings and identify further *MAPT* and *BAG3* associated CpGs which may indicate causal relationships and contribute to biological pathways. A further study included in our review which identified MAPT within analysis was Go *et al*. [65]. Go *et al*. [65] investigated plantation work and self-reported organochlorine pesticide exposure in blood and postmortem brain samples of Parkinson’s disease patients, including identifying pesticide presence in brain tissue [65]. Individual CpGs were identified for both blood and brain and pathway analysis were also performed. On comparing ≥10 years plantation work to 0 years, identified DML were mapped to genes and pathways. MAPT was identified among the most important genes in mapped pathways further suggesting the investigation of DNAm profiles and *MAPT* expression, including its role in biological pathways is warranted.

Huen '*et al*.’s [54] study was the only study investigating children (<18 years of age). Huen *et al*. [54] did not identify any statistically significant associations between blood-derived *PON1* DNAm profiles, children’s cognitive outcomes at aged 7 years measured with the WISC-IV and organophosphate pesticides. Huen *et al*.’s [54] supplementary material does however provide insights including statistical evidence of hypermethylation of *PON1* site 3 (effect −0.08, 95% CI: −0.14, −0.01) and site 7 (effect −0.07, 95% CI −0.12, −0.01) with lower prenatal diethyl phosphate exposure. These data may suggest further work analyzing *PON1* may provide us with insights into the underlying biology behind pesticide exposure.

Other literature has been investigating the impact of pesticides exposure on methylation profiles and cognition. Chiu et al., [93] investigated chloropyrifos (pesticide) exposure, DNAm and children’s cognitive outcomes using the cognitive domain of the CDIIT [93]. Chiu et al., [93] did not identify any statistically significant associations between DNAm and the cognitive test domain (crude p value = 0.26, adjusted p= 0.96). However, chloropyrifos exposure was associated with lower performance in the cognitive test domain in multivariable models (adjusted B −1.2 (−2.23, −0.15) p value = 0.025). Chloropyrifos exposure was also associated with increased *PPARy* DNAm (crude p = 0.027, adjusted p = 0.032) [93]. Chiu et al., [93] identified associations between pesticide exposure and cognitive function and between pesticide exposure and DNAm. However, this study did not undertake analysis which associated all three of these variables together and according to our eligibility criteria (Table 1) was not included in our evidence synthesis. A recent systematic review and meta-analysis by Rohr *et al*. [94] discuss the epigenetic impact of pesticides on human epigenetics including DNA methylation [94]. This study provides a comprehensive overview of current findings; however, it does not discuss relationships between epigenetic changes and cognition.

### Insights from literature on other environmental exposures

We identified studies in this review which investigated differential DNAm profiles and cognitive outcomes in response to air pollution, heavy metals, chemicals, and pesticides exposure. We did not identify any studies that met our eligibility criteria which investigated noise pollution, light pollution, green or blue space, walkability, cyclability, or more novel exposures. However, other studies within the field can provide us with some insights on these other exposures.

Literature has, e.g., highlighted the impact of chemicals which may circulate in the environment from industrial production such as formaldehyde. Formaldehyde is a volatile organic compound released as a result of forest fires or wildfires [95]. In Tong *et al*. [96] AD case/control study, endogenous formaldehyde concentrations were estimated from postmortem cortical samples and from urine samples in small cohorts [96]. When looking at urine formaldehyde estimates, concentrations were higher in participants with abnormal cognition compared to controls when cognition was measured using the Clinical Dementia Rating (0.042 vs. 0.002 mM). Urine formaldehyde levels were positively correlated with severe degrees of dementia in AD patients (*r* = 0.933). Tong *et al*. [96] also found that hippocampal formaldehyde concentration correlated negatively with global DNAm levels (*r* = −0.949) in postmortem samples [96]. This study focused on endogenous formaldehyde and did not enquire into potential exogenous sources of formaldehyde exposure, thus not directly relating to environmental exposure and so was not included in the present study. However, this study does highlight the ability to explore more novel exposures such as forest/wildfires through potentially using high-performance liquid chromatography with fluorescence to estimate formaldehyde concentrations in human samples.

Literature has also provided some insights into green space exposure. Dockx *et al*. [97] assessed placental DNAm profiles from mother/infant pairs against maternal exposure to green space through calculating percentages of green spaces surrounding participant’s homes in buffers [97]. Dockx *et al*. [97] found that those with nature within a 50 m and 100 m proximity to residence had a 3% (95% CI 1.09, 4.91) and 1.98% (95% CI 0.28, 3.68) higher *HTR2A* methylation compared to those without. However, authors of this study noted that the cohort region within Belgium has one of the highest percentages of green space in Belgium, and so this study may not be representative of the general Belgian population. *HTR2A* is a serotonin receptor associated with neurodevelopment in children and cognitive function in adults [98]. Dockx *et al*. [97] study is a useful example of how previous literature is useful for the expansion of our knowledge of cognitive outcomes from environmental exposures associated with underlying DNAm.

These literature examples demonstrate that the field has been exploring the associations between the environment and DNAm profiles. However, future research on these identified DNAm and disease outcomes, including neurodegenerative disease, will enhance our understanding of this biological connection between environmental exposures and cognitive impairment/neurodegenerative disease.

### Strengths and limitations

This review includes studies identified from an extensive systematic literature search on the environment, DNAm, and cognition. We were interested in a range of environmental exposures and screened recent relevant review study reference lists to provide us with additional studies. We additionally discussed in this review, studies which assessed the impact of environmental exposures on DNAm without associating this to a cognitive outcome to provide a summary of additional epigenetic based findings and environmental exposures which are being investigated within research.

This review focuses on population-based studies as we were interested in identifying epigenetic markers located from human study. This review also focuses on one type of epigenetic modification, DNAm. Non-coding RNAs and histone modifications are further epigenetic modifications which may be of use in examining the environmental–neurodegenerative relationship. Utilizing a similar search strategy and methodology of this review, summarizing any available evidence on the impact of the environment on neurodegenerative disease through histone modifications and non-coding RNAs in the future would be a benefit to the overall research field.

We conducted risk of bias assessments on all studies included in this review, utilizing different assessment tools for cohort and cross-sectional studies and additionally utlizing the AACODS checklist for Zhenjiang *et al*. [55] and Casazza *et al*. [66] preprint studies. Through including preprint articles in our review, we provided a more comprehensive summary of the current field including recent unique analysis, enhancing our review.

The environmental descriptions provided within our extraction tables are somewhat lacking in description. We note that more comprehensive descriptions would be beneficial to create a better understanding of the specific types of exposures and their measurements; therefore, we have included a recommendation for further study. Similarly, we were unable to provide descriptions of the environment, cognitive function, cognitive impairment, or neurodegenerative disease from included studies as these were not stated. We provided definitions of these for our review; however, we were unable to assess if included studies worked on similar descriptions.

### Methodological guidance for future study

**Figure 2.**
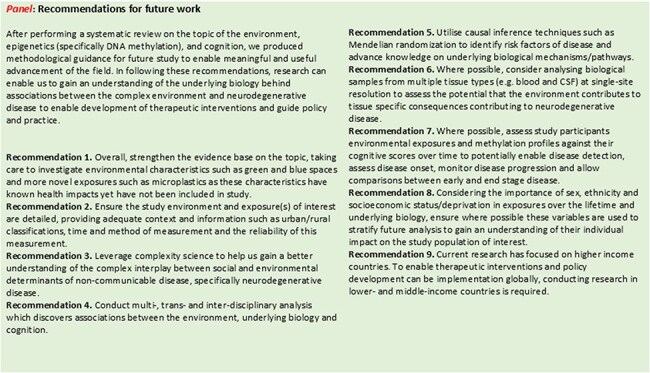
Panel of recommendations for future research.

Based on the findings of this review, we propose recommendations to inform the design, conduct, and reporting of future study within the field (see Fig. 2 for panel recommendations).

#### Environment and complexity

Studies included in this review assessed the impact of pollutants relating to air, water, and soil pollution, namely heavy metals, pesticides, and other chemicals. We did not identify any study meeting our eligibility criteria, which investigated associations between noise pollution, light pollution, green space, blue space, walkability, cyclability, or novel exposures such as microplastics or forest fires, DNAm, and a cognitive outcome. There is an increasing interest in green and blue spaces, e.g., in both mental health and brain health research, with the potential to use these are a type of therapeutic intervention, “prescribing” time in these spaces to promote healthy brain aging [50, 99, 100]. Microplastics and forest fires are additional exposures which we are becoming increasingly aware of globally, noting the possible implications of these on our health [101–104]. Considering the small amount of literature we identified in the current field, further research of all environmental exposures is required; however, care should be taken to ensure all exposures are evaluated in correspondence to methylation profiles and cognitive outcomes.

In addition, there is a need for future work to leverage and build on current complexity science such as a the CLD developed by Avila-Palencia *et al*. [49], exploring causal mechanistic pathways between the urban environment and cognitive decline, ensuring that the inter-relationships between and interactions of environmental exposures are evaluated with regards to cognitive outcomes. To begin incorporating this into future work, studies could assess co-exposures or co-presence, the benefits/necessities of which have been highlighted in syndemics literature [105, 106]. Traffic-related pollution for example, directly relates to air pollution and noise pollution [107, 108] and can have indirect impacts of soil and water pollution [12, 109]. Therefore, when interested in traffic-related pollution as an exposure within a study, utilizing available data on all these pollutants with appropriate statistical techniques could add further evidence to the field and ensure that potential interactions can be assessed. It is also important for further studies to provide comprehensive descriptions of the environmental exposures to be assessed. As we previously discussed, we were not able to provide detailed descriptions of some exposures included in our review. Ensuring detailed accounts of the potential local sources of environmental exposures, their quantification or measurement, and reporting participant’s time lived at current address for example, may aid our interpretations of results in future studies. Additionally, we were unable to discuss the impact of urban vs. rural living in this review considering current studies did not specify a particular interest in or focus on urban, suburban, peri-urban, or rural populations. Considering these classifications in future work will allow comparisons to be drawn between the exposure risks in different environments.

We identified literature that assessed the impact of environmental exposure on epigenetic markers. Authors of these studies often discussed identified markers relationship with cognition or neurodegenerative disease using knowledge from literature. Although these studies were not eligible for inclusion in this review considering the lack of formal association analysis, this research does highlight that epigenetic markers are being associated with environmental exposures. However, it is crucial that future work formally associates identified markers with cognitive outcomes to further our understanding of the environmental-neurodegenerative relationship. Performing mechanistic reviews is a potential way to contri–ute to this understanding. Mechanistic reviews systematically search the literature for exposure–mechanism studies and mechanism-outcome studies separately [110]. This information and knowledge will allow us to plan research effectively to validate the current findings and the bridge our knowledge gaps.

One study included in our review, Casazza *et al*. [66] performed a type of causal analysis, Mendelian randomization. Mendelian randomization is a technique which may be used to explore the environmental–neurodegenerative relationship outlined in Glover *et al*. [12]. Briefly, statistically significant CpG sites identified in response to environmental exposures can be used to generate a list of genetic variants/SNPs using a database (such as http://www.godmc.org.uk/resources.html) comprised of genetic variants that have already been associated with differential methylation at CpG sites. These genetic variants then act as a proxy to assess the causal relationship between differential methylation at particular CpG sites and a disease outcome. Future work should consider performing causal analysis in addition to association analysis. These approaches combined will advance knowledge of the underlying relationships and mechanisms between the environment, underlying biology, and neurodegenerative disease.

#### DNAm

Studies included in this review assessed blood-derived or postmortem brain-derived methylation profiles. Peng *et al*. [57] compared blood- and brain-derived methylation profiles to assess *LAMB2*-related methylation sites identified within their study. The methylation profiles derived from blood samples differed from those derived from postmortem adult brain samples, suggesting methylation was tissue specific. To further assess if methylation sites are tissue-specific, future work, where possible, would benefit from assessing samples from multiple organ systems With regard to environment–neurodegenerative work, assessing brain/central nervous system methylation is important. However, a challenge which accompanies postmortem samples for methylation data is the potential degradation of DNA [111, 112], creating issues of accuracy and reliability. Additionally, postmortem samples may not reflect the dynamic changes in DNAm which occur during the progression of disease [113]. A potential alternative for assessing central nervous system DNAm is cerebrospinal fluid (CSF) as suggested by Paul *et al*. [64]. CSF may provide a minimally invasive way to assess methylation profiles as patients age. CSF samples may provide insights of early-stage disease development and enable monitoring of disease progression from repeated sample collection. Monitoring environmental exposures over time and changing CSF-derived methylation values in diseased patients could be a future goal in the field.

Numerous studies included within this review assessed global methylation, providing a broad overview of the epigenetic consequences of environmental exposures. However, it may be more beneficial for future research to concentrate on individual methylation sites where possible. In non-communicable diseases including neurodegenerative disease, small alternations at specific sites can have great impacts on underlying biology and play crucial a role in pathogenesis. Through identifying specific DNAm sites associated with neurodegenerative disease, we can uncover epigenetic signatures which have potential to serve as biomarkers (with replication of findings and significance) and aid us in developing therapeutic interventions. Identification of individual DNAm sites is also important for providing a basis for biological pathway implications. Around half of the studies included in this review performed a type of biological pathway analysis to assess how environmental exposures were impacting underlying biology to influence cognitive outcomes. There is currently a small amount of concrete evidence on the biological pathways underpinning the associations between the environment, epigenetics, and neurodegenerative disease outlined in Glover *et al*. [12]. In order to advance our knowledge of potential pathways, future research should strive toward identifying individual methylation sites and where possible perform pathway analysis.

#### Cognition

Included studies in this review assessed the impact of an environmental exposure on the methylation profiles of either adults or children, relating these profiles to cognitive impairment or neurodegenerative disease or cognitive function respectively. However, few adult studies included in this review stratified their identified DNAm sites by their cognitive outcome (e.g. stratification by CASI score, Braak stage), instead often opting for direct association to PD. While the analysis these studies have carried out is important for us to gain an understanding of general methylation associations with neurodegenerative disease, stratifying methylation sites by cognitive scores which are often evaluated within studies, would give the added benefit of potentially correlating methylation sites to a disease stage. In our screening process, we identified studies which investigated an environmental exposure, DNAm and neurodevelopmental outcomes including behavior [114–117]. These studies provide evidence that environmental exposure is impacting DNAm profiles and have consequences on infant, children, and adolescent brain function. It would be beneficial to extend this work through longitudinal follow up, and where possible assess if early-life neurodevelopment is indicative of future cognitive deficits or impairment.

#### Sub-analysis and risk of bias

No study included in this review stratified their outcomes by ethnicity or participant deprivation status, and few studies stratified by sex. Sex, ethnicity, and deprivation/socioeconomic status are variables of significant importance considering research indicates variations in methylation profiles in response to these factors [118–120]. Neurodegenerative diseases such as AD is often reported to have a higher incidence in women [5] and PD is reported to have a higher incidence in men [121], suggesting there may underlying biological influence in disease susceptibility. Future study should consider stratification by sex, ethnicity, and socioeconomic status when statistically possible. All studies in this review took place in upper-middle or high-income status countries according to the World Bank classification system. Considering the variability in environmental exposures, race and ethnic diversity across different regions worldwide, future research should aim to expand to countries of a lower-income status. This will give us an understanding of how environmental exposures impact methylation profiles in diverse populations and will ensure research findings will inform the development of relevant therapeutic interventions.

All studies included in this review underwent a risk of bias assessment. These assessments demonstrated that current research consistently provides detailed descriptions of statistical methods and a comprehensive evaluation of study findings. However, future research should ensure that methodologies are clearly reported, specifying which laboratory samples were stored and quantified within. Additionally, methods for handling outlying or missing data should be described. In future longitudinal studies, it is important to discuss any loss to follow-up and describe techniques used to address this. Developing standards relating to the detailed composition of datasets and study methodologies may allow an easier comparison of work. Future study being of a longitudinal nature would be beneficial for this field, potentially enable the identification of early cognitive impairment or neurodegenerative disease and assessing the progress of the condition over time in response to continued environmental exposure. In future work involving young participants, assessing their cognitive function over time may provide us with beneficial insights on the early brain from infancy through adolescence to adulthood.

## Conclusion

This review synthesises the small number of population-based studies which have examined the association between environmental exposures, DNAm and cognitive outcomes including neurodegenerative disease. Evidence indicates that environmental factors air pollution (PM2.5), proximity to roads, heavy metals (lead, aluminum, manganese, and mercury), and pesticides (organophosphates and organochlorines) influence human methylation profiles with consequences on cognitive outcomes. Validation of key methylation sites, genes of interest, and potential biological pathways identified in current study is required. Environmental exposures including green space, blue space, noise pollution, light pollution, and more novel exposures such as microplastics have currently not been investigated. Future research should adopt a complexity science approach including causal investigation to explore interrelationships between environmental factors, underlying biology and cognition. This approach will provide a more comprehensive understanding of the broader picture and underlying biological pathways. Performing mechanistic reviews may also allow us to bridge the gap between exposure–mechanism and mechanism–outcome literature. Examining the impact of socioeconomic status, ethnicity and sex has been largely absent in previous research, a factor to consider in future research outlined in our methodological recommendations. Advancing the field through provided recommendations is hoped to provide a basis of knowledge to inform practice, policy and decision making related to environmental planning, development, policy, and public health interventions aimed at promoting healthier brain aging.

## Supplementary Material

dvae027_Supp

## Data Availability

Data are available in the supplementary files or from the corresponding author on request. No primary data were generated in this study.
