# Supplementary material for: A systematic review of associations between the environment, DNA methylation, and cognition
Source: Environ Epigenet. 2024 Dec 16;11(1):dvae027. doi: 10.1093/eep/dvae027 (PMC11776599; doi:10.1093/eep/dvae027)
Supplement: dvae027_Supp [file dvae027_supp.zip › dvae027_Supp/Supplementary Material FINAL.docx]

## **Section 1.**

## **Search strategy**

Databases:

MEDLINE

Embase

PSYCHInfo

PubMed

Epigenetic terms:

epigenetic*

epigenomic*

DNA modification

CpG

DNA methylation

hypermethylation

hypomethylation

gene modification

gene expression

Urban environment terms:

urban adj1 environment

green adj1 space

blue adj1 space

pollut*

air pollution

air adj1 quality

PM

particulate adj1 matter

nitrogen dioxide

sulfur dioxide

sulphur dioxide

carbon monoxide

ozone

noise pollution

soil pollution

soil adj1 quality

soil adj1 composition

soil toxin*

water pollution

water adj1 quality

heavy metal*

arsenic

cadmium

chromium

copper

mercury

light pollution

streetlight

street light

walkability

pedestrian adj1 infrastructure

cyclability

bikeability

cycling adj1 infrastructure

bicycle adj1 infrastructure

road*

traffic

heat adj1 island

heatwave*

heat wave*

coldwave*

cold wave*

flood*

pesticide*

wildfire*

wild fire*

forest fire*

microplastic*

micro plastic*

climate

ambient adj1 temperature

heat adj1 stress

excess adj1 heat

Cognitive terms:

cognition

cognitive*

mild adj1 cognitive impairment

brain*

dementia

neurodegenerative adj1 disease

neurodegeneration

Alzheimer*

Parkinson*

OR was used to link terms of the same theme (themes being epigenetic, environment and cognitive)

AND was then used to link the three main themes together

Searches from database start to September 2023

Limits on the search: Human studies, English language

Inclusion Criteria:

1. Studies which identified DNA methylation sites associated with an outdoor characteristic of the urban environment (built, natural or physical) which is linked to cognitive impairment or neurodegenerative disease through association analysis

2. Studies from any world region

3. Pre-prints

Exclusion Criteria:

1. Studies which identified genetic markers but not epigenetic markers

2. Studies focused on indoor urban environment characteristics

3. Studies in which DNA methylation sites were not linked to cognitive impairment or neurodegenerative disease (no association analysis)

4. Review studies reference lists will be assessed for suitable studies but not included in the data extraction process

5. Cell culture/in-vitro

6. Conference abstracts

7. Dissertations or theses

8. Animal studies

9. Studies focused on neurodevelopmental or mental illness outcomes – ASD, schizophrenia, bipolar disorder etc

Databases:

BioRxiv

MedRxiv

Search terms:

Epigenetic AND

Urban environment AND

Cognitive impairment OR neurodegeneration

Limits on search: within the last year (September 2022- September 2023)

## **PICO**

| Participants | Intervention | Comparison | Outcome |
| --- | --- | --- | --- |
| - Any age or sex and from any country. - Human (Cell culture/in-vitro studies were not included in this review) - Exposed to environmental exposure(s) of interest | Studies had to be investigating at least one of the following characteristics of the environment:   - Outdoor air pollution including particulate matter, ozone, sulfur oxides, nitrogen oxides, carbon oxides, heavy metals - Outdoor soil pollution including heavy metals, chemicals or pesticides - Outdoor water pollution including heavy metals, phenols or other chemicals - Outdoor noise pollution from sources including transport, industry, construction - Outdoor light pollution focused on outdoor light at night and streetlight density - Green and blue space access and quality including parks, greenways, public gardens, public open space, park rivers or lakes, trees and low-lying vegetation - Neighbourhood walkability measured by land use mix, urban green space presence, pedestrianisation, traffic density, sidewalk density - Neighbourhood cyclability (bikeability) measured by cycle lane availability - Heat/temperature measures by heatwave statistics or longitudinal temperature measurement - Novel exposures/pollutants including microplastics, wildfires and forest fires | - No comparator or control groups were required - Studies were included if they included a control group (a group of participants exposed to the environmental exposure with healthy cognitive function) | - Identified epigenetic markers (DNA methylation) by quantitative methods which associated an environmental exposure of interest with cognitive function, cognitive impairment and/or neurodegenerative disease through analysis - Studies which specifically used testing of neurodevelopmental outcomes as opposed to cognitive outcomes or used mental health outcomes were not included in this review |

## **Section 2.**

## **Definitions**

**Table. Definitions of main concepts**

| **Concept** | **Definition** |
| --- | --- |
| Environmental exposures | Various aspects of the environment which can impact the health and well-being of populations. In this review we mainly focused on physical and chemical factors. |
| Urban environment | Towns and cities with high density populations and surrounding sub-urban and peri-urban areas between urban and rural settlements |
| Cognitive function | The capacity for the brain to carry out executive functions to allow attention, memory, judgement, learning, thinking, and decision making |
| Cognitive impairment | Decline of executive function impacting attention, memory, judgement, learning, thinking and decision making |
| Neurodegenerative disease | The progressive decline of brain structure and function from neuronal death producing cognitive impairment |

## **Section 3.**

| DOI | Author | Hypothesis/Objective | Study population selection | Study population representativeness | Study population characteristics | Statistical analysis | Interpretation and eval of results | Study limitations | Specimen characteristics and assay methods | Lab measurement | Biomarker data modelling | Score |
| --- | --- | --- | --- | --- | --- | --- | --- | --- | --- | --- | --- | --- |
| Cross-sectional : BIOCROSS | | | | | | | | | |  |  |  |
| <https://doi.org/10.1016/j.parkreldis.2015.01.007> | Nielsen et al., (2015) | 2 | 1 | 1 | 1 | 2 | 1 | 1 | 1 | 1 | 0 | 11 |
| <https://dx.doi.org/10.1097/JOM.0000000000000474> | Yang et al., (2015) | 2 | 2 | 1 | 1 | 2 | 2 | 0 | 1 | 0 | 1 | 12 |
| <https://doi.org/10.1016/j.scitotenv.2018.07.143> | Paul et al., (2018) | 1 | 1 | 1 | 1 | 2 | 2 | 0 | 1 | 0 | 1 | 10 |
| <https://doi.org/10.1007/s12640-017-9736-7> | Castillo et al., (2017) | 1 | 1 | 1 | 1 | 1 | 1 | 0 | 1 | 0 | 1 | 8 |
| <https://doi.org/10.1186/s12868-020-00582-4> | Go et al., (2020) | 1 | 1 | 1 | 1 | 2 | 2 | 1 | 1 | 0 | 0 | 10 |
| <https://doi.org/10.1186/s13148-021-01051-3> | Paul et al., (2021) | 1 | 1 | 1 | 1 | 2 | 2 | 1 | 1 | 0 | 0 | 10 |
| <https://doi.org/10.1021/acs.est.0c01696> | Wan et al., (2021) | 2 | 1 | 1 | 1 | 2 | 2 | 0 | 1 | 0 | 0 | 10 |
| Cohort : Joanna Briggs | | | | | | | | | |  |  |  |
| DOI | **Author** | **Study recruitment** | **Exposure measure** | **Exposure validity and reliability** | **Confounding** | **Adjustment** | **Free from outcome** | **Outcome measurement** | **Follow-up time** | **Follow-up loss** | **Addressing incomplete follow-up** | **Statistical analysis** |
| <https://doi.org/10.3389/fgene.2022.871820> | Guo et al., (2022) | Y | Y | Y | Y | Y | U | Y | Y | Y | N/A | Y |
| <https://doi.org/10.1016/j.envint.2018.08.044> | Huen et al., (2018) | Y | Y | Y | Y | Y | U | Y | Y | N | N | Y |
| <https://doi.org/10.1038/s41598-017-00384-5> | Cardenas et al., (2017) | Y | Y | Y | Y | Y | U | Y | Y | N | N | Y |
| <https://doi.org/10.1289%2FEHP2034> | Peng et al., (2018) | Y | Y | Y | Y | Y | U | Y | Y | Y | N/A | Y |
| <https://doi.org/10.1016/j.envint.2016.12.009> | Lee et al., (2017) | Y | Y | N | Y | Y | U | Y | Y | N | N | Y |
| Pre-print: AACODS | | | | | | | | | | | | |
| DOI | **Author** | **Authority** | **Accuracy** | **Coverage** | **Objectivity** | **Date** | **Significance** |  |  |  |  |  |
| <https://doi.org/10.1101/2023.06.30.23292085> | Zhenjiang et al., (2023) | The authors are associated with reputable organisations, hold professional qualifications and have sufficient experience in this field. Other works produced by the authors have been cited by other academics. There is a detailed reference list in this article. | The aim and methodology have been clearly stated and met/adhered to. This study has not been peer reviewed. Data collected in this study is explicit and appropriate for the research carried out. | Limitations were clearly discussed. This study spanned the fields of epigenetics, neurodegenerative disease and the environment. | The study appears balanced and objective. | This study was posted in June 2023. Brain samples used in this study were gathered from 2007 onwards. References used are contemporary. | The research is meaningful and expands current knowledge in this research area, which is limited. By identifying DNA methylation changes associated with PM2.5 exposure in samples from AD patients, it demonstrated the need for future research in this field. |  |  |  |  |  |
| <https://doi.org/10.1101/2023.07.21.23293014> | Casazza et al., (2023) | The authors are associated with reputable organisations. The authors are part of a research group investigating AD (DIG-PD), which is part of a larger neurodegenerative disease research network. Other work in this field has been produced by the authors. There is a detailed reference list. | The study has not been peer reviewed. The methodology has been clearly outlined and is adhered to. The research aim is clearly stated and met. | The study investigated the relationship between epigenetics, PD and the environment using a cohort of French agricultural workers ages 18 - 75, utilising a case-control study design. | The study was reported in an objective manner. | This study was posted in July 2023. PD patients in the DIG-PD study were recruited between May 2009 and July 2013, and were followed up annually for 7 years, however only clinical patient data collected by physicians during recruitment was used in this study. | This research is meaningful and expands current knowledge in this field. It follows a complex and thorough methodology using multiple cohorts and incorporates causal analysis. |  |  |  |  |  |

**BIOCROSS Evaluation tool – mentioning all feature of a domain (score of 2), mention of at least 1-2 (score of 1), mention of none (score of 0)**

Item, Issues to consider (IC), Study quality feature

, 1st Domain: Study rational,

1., 1.1 Was the biomarker under study described?1.2 Was the rationale for the study (research question) clearly presented?1.3 Were the study objectives/ hypothesis clearly stated?, Hypothesis/Objective

, 2nd Domain: Design/Methods,

2., 2.1 Were the characteristics of the study participants presented?2.2 Were the disease stages or comorbidities of the included participants described?2.3 Were the inclusion and exclusion criteria for study participation defined?, Study population selection

3., 3.1 Was the sampling frame reported (study population source)3.2 Was the participation rate reported (i.e. eligible persons at least 50%)?3.3 Was sample size justification or power description provided?, Study population representativeness

, 3rd Domain: Data analysis,

4., 4.1 Were the study population characteristics (i.e. demographic, clinical and social) presented?4.2 Were the exposures and potential confounders described?4.3 Were any missing values and strategies to deal with missing data reported?, Study population characteristics

5., 5.1 Did the authors clearly report statistical methods used to calculate estimates (e.g. Spearman/Pearson/Linear regression, etc.)?5.2 Were key potential confounding variables measured and adjusted statistically in reported analyses?5.3 Was the raw effect size estimate (correlation coefficient, beta coefficient) or measure of study precision provided (e.g. confidence intervals, precise (!) p-value*)?, Statistical analysis

, 4th Domain: Data interpretation,

6., 6.1 Was the data discussed in the context of study objectives/hypotheses?6.2 Was the interpretation of the results considering findings from similar studies?6.3 Was the biological context described?, Interpretation and evaluation of results

7., 7.1 Was the cross-sectional nature of the analysis discussed?7.2 Did the authors acknowledge restricted interpretation due to measurements at one point in time and no statement about causality possible using cross-sectional studies?7.3 Did the authors acknowledge need for consistency with other research?, Study limitations

, 5th Domain: Biomarker measurement,

8., 8.1 Were the measurement methods described? (assay methods, preservation and storage, detailed protocol, including specific reagents or kits used)8.2 Were the reproducibility assessments performed for evaluating biomarker stability?8.3 Were the quantitation methods well described?, Specimen characteristics and assay methods

9., 9.1 Was the laboratory/place of measurement mentioned?9.2 Were any quality control procedures and results reported (e.g. reported coefficient of variation?9.3 Were the analyses blinded for laboratory staff?, Laboratory measurement

10., 10.1 Was the distribution of biomarker data reported (if non-normal how it was standardized)?10.2 Did the authors report on methods or outlier detection and handling?10.3 Were any possible errors resulting from measurement inaccuracies discussed?, Biomarker data modeling

*Reporting not significant (ns) or p > 0.05 is not precise and does not allow a judgment on precision

**Joanna Briggs Cohort checklist**

1. Were the two groups similar and recruited from the

same population?

2. Were the exposures measured similarly to assign

people to both exposed and unexposed groups?

3. Was the exposure measured in a valid and reliable

way?

4. Were confounding factors identified?

5. Were strategies to deal with confounding factors

stated?

6. Were the groups/participants free of the outcome

at the start of the study (or at the moment of

exposure)?

7. Were the outcomes measured in a valid and reliable

way?

8. Was the follow up time reported and sufficient to

be long enough for outcomes to occur?

9. Was follow up complete, and if not, were the

reasons to loss to follow up described and explored?

10. Were strategies to address incomplete follow up

utilized?

11. Was appropriate statistical analysis used?

AACODS

Authority

Identifying who is responsible for the intellectual content. Individual author: • Associated with a reputable organisation? • Professional qualifications or considerable experience? • Produced/published other work (grey/black) in the field? • Recognised expert, identified in other sources? • Cited by others? (use Google Scholar as a quick check) • Higher degree student under “expert” supervision? Organisation or group: • Is the organisation reputable? (e.g. W.H.O) • Is the organisation an authority in the field? In all cases: • Does the item have a detailed reference list or bibliography?

Accuracy

• Does the item have a clearly stated aim or brief? • Is so, is this met? • Does it have a stated methodology? • If so, is it adhered to? • Has it been peer-reviewed? • Has it been edited by a reputable authority? • Supported by authoritative, documented references or credible sources? • Is it representative of work in the field? • If No, is it a valid counterbalance? • Is any data collection explicit and appropriate for the research? • If item is secondary material (e.g. a policy brief of a technical report) refer to • the original. Is it an accurate, unbiased interpretation or analysis? Archived at the Flinders Academic Commons: <http://dspace.flinders.edu.au/dspace/>

Coverage

All items have parameters which define their content coverage. These limits might mean that a work refers to a particular population group, or that it excluded certain types of publication. A report could be designed to answer a particular question, or be based on statistics from a particular survey. • Are any limits clearly stated?

Objectivity

It is important to identify bias, particularly if it is unstated or unacknowledged. • Opinion, expert or otherwise, is still opinion: is the author’s standpoint clear? • Does the work seem to be balanced in presentation?

Date

For the item to inform your research, it needs to have a date that confirms relevance • Does the item have a clearly stated date related to content? No easily discernible date is a strong concern. • If no date is given, but can be closely ascertained, is there a valid reason for its absence? • Check the bibliography: have key contemporary material been included?

Significance

This is a value judgment of the item, in the context of the relevant research area • Is the item meaningful? (this incorporates feasibility, utility and relevance) • Does it add context? • Does it enrich or add something unique to the research? • Does it strengthen or refute a current position? • Would the research area be lesser without it? • Is it integral, representative, typical? • Does it have impact? (in the sense of influencing the work or behaviour of others)
